# Advanced applications of synthetic biology technology in biosynthesis of bioactive compounds from medicinal plants

**DOI:** 10.1016/j.chmed.2025.11.006

**Published:** 2025-11-16

**Authors:** Yingjun Liu, Anying Ji, Haiyang Jia, Huan Sun

**Affiliations:** a Institute of Medicinal Plant Development, Chinese Academy of Medical Science & Peking Union Medical College, Beijing 100193, China; b School of Chemistry and Chemical Engineering, Beijing Institute of Technology, Beijing 100081, China

**Keywords:** bioactive compounds, medicinal plants, metabolic engineering, regulatory tools, synthetic biology

## Abstract

Medicinal plants serve as valuable sources of bioactive compounds with critical applications across pharmaceutical, agricultural, and industrial sectors. Compared to chemical synthesis and plant extraction, synthetic biology offers a green, efficient, and sustainable alternative for producing bioactive compounds, which represents a state of art technology. However, this technology still faces several challenges, including overly long metabolic pathways, inadequate catalytic efficiency of key enzymes in the pathway, and incompatibility between gene elements and host cells, leading to low yields of target bioactive compounds. The development and application of regulatory tools in synthetic biology hold great promise for overcoming these obstacles. This review first summarizes the classification and biosynthesis of bioactive compounds based on structural types. Subsequently, recent advancements are outlined in regulation tools and their application in the heterologous production of bioactive compounds. This review aims to establish a foundation for the efficient production of bioactive compounds based on microbial cell factories. This not only has significant practical implications for reducing the resource consumption and environmental impact of traditional production methods, but also highlights the central role of synthetic biology in promoting the sustainable production of bioactive compounds derived from medicinal plants.

## 1 Introduction

**Fig. 1:**
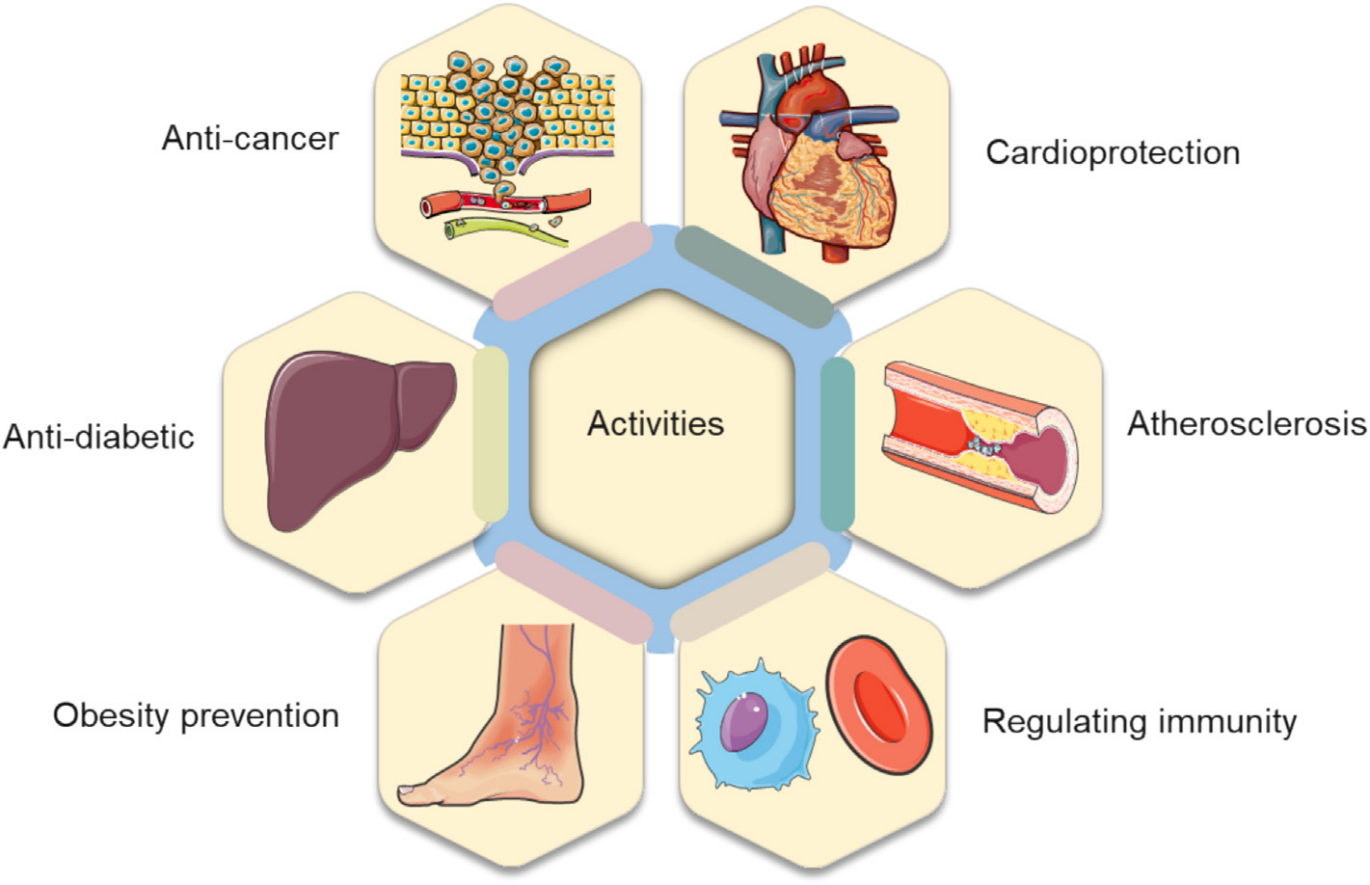
Common activities of bioactive compounds in medicinal plants.

Medicinal plants produce diverse bioactive compounds with significant pharmacological properties (Fig. 1) (Li et al., 2023). Among these, flavonoids, terpenoids, and alkaloids constitute the three major groups of these compounds (Wang et al., 2019, Zhao et al., 2025). While non-essential for the plant’s survival, these compounds greatly benefit human health (Panchal and Tiwari, 2017, Adedeji and Babalola, 2020), as demonstrated by examples like scutellarin’s neuroprotection (Sheng et al., 2023), berberine’s anti-inflammatory effects (Wei et al., 2023), and the neuroprotective potential of goji berry and ashwagandha extracts (Zhang & Wang, 2025). Widely used in pharmaceuticals, nutraceuticals, pesticides, cosmetics, and fine chemicals (Cravens et al., 2019, Taroncher et al., 2021), they include clinically important drugs like artemisinin, morphine, paclitaxel, ginsenosides, lovastatin, and tanshinone (Buranasudja et al., 2021, Kowalczyk et al., 2024), and icaritin, approved in China in 2022 for liver cancer (Wang, Wang, Liu, Yang, Zhang, & Kong, 2023). The high demand is expected to grow, the flavonoid market alone is projected to rise from $149.77 million (2020) to $271.78 million by 2030 (Li et al., 2022).

Most bioactive compounds are obtained through plant extraction, including crocetin, glycyrrhizin, icaritin, salidroside, rosavin and so on (Qiu et al., 2022, Xia et al., 2022, Hong et al., 2023, Guo et al., 2024, Wu et al., 2025). However, low concentrations in medicinal plants lead to high raw material consumption, and plant cultivation is time-consuming due to time, space, and climate, potentially threatening food security (Nomura et al., 2019, Wang et al., 2021, Shan et al., 2023, Zhou et al., 2024, Li et al., 2024). Chemical synthesis often suffers from long reaction pathways, complex reaction conditions, low product yields, high production costs, and generation of environmentally harmful by-products (Marchev et al., 2016, Nose et al., 2019, Wang et al., 2021, Xu et al., 2021, Li et al., 2024, Zhou et al., 2023). These limitations hinder stable supply of bioactive compounds, necessitating development of novel, cost-effective, resource-efficient, and safe production methods. Fortunately, synthetic biology has emerged as a highly promising production model due to its environmentally friendly, low-carbon, and sustainable attributes (Parkan et al., 2014, Yao et al., 2019).

Synthetic biology represents a novel and rapidly developing cross-disciplinary area within biotechnology (Glaser, 2016, Cravens et al., 2019, Jamieson et al., 2021). By integrating systems biology, chemistry, computer science, and genetic engineering technology, it aims to design, simulate, and construct novel artificial biological systems or modify existing systems to perform innovative functions (Jiang et al., 2024). Despite challenges from complex interactions requiring iterative optimization following the design-build-test-learn cycle, significant progress has been made in this field. For example, studies have demonstrated that engineered expression systems combined with metabolic engineering in yeast achieved 448.64 mg/L sanguinarine production (Gou et al., 2024). Increased sanguinarine production, driven by its antimicrobial and antineoplastic activities, will expand its applications. In conclusion, synthetic biology offers a solution for producing bioactive compounds, addressing the supply–demand imbalance.

Advances in genomics and proteomics have clarified biosynthetic pathways of bioactive compounds in medicinal plants. A prime example is the detailed analysis of the paclitaxel biosynthetic pathway. Notably, paclitaxel, a pivotal anti-cancer drug, faced significant limitations due to the scarcity of plant resources and the complexity of its chemical synthesis. In this context, research successfully identified key missing enzymes enabling heterologous baccatin III production (Jiang et al., 2024). Synthetic biology now enables the transfer of plant pathways to microbial hosts for efficient production of plant-derived drugs like artemisinic acid and vinblastine in engineered yeast (Gao et al., 2023, Paddon et al., 2013). This method not only addresses the challenge of obtaining natural products but also establishes heterologous biosynthesis as a critical research area in current scientific exploration.

**Fig. 2:**
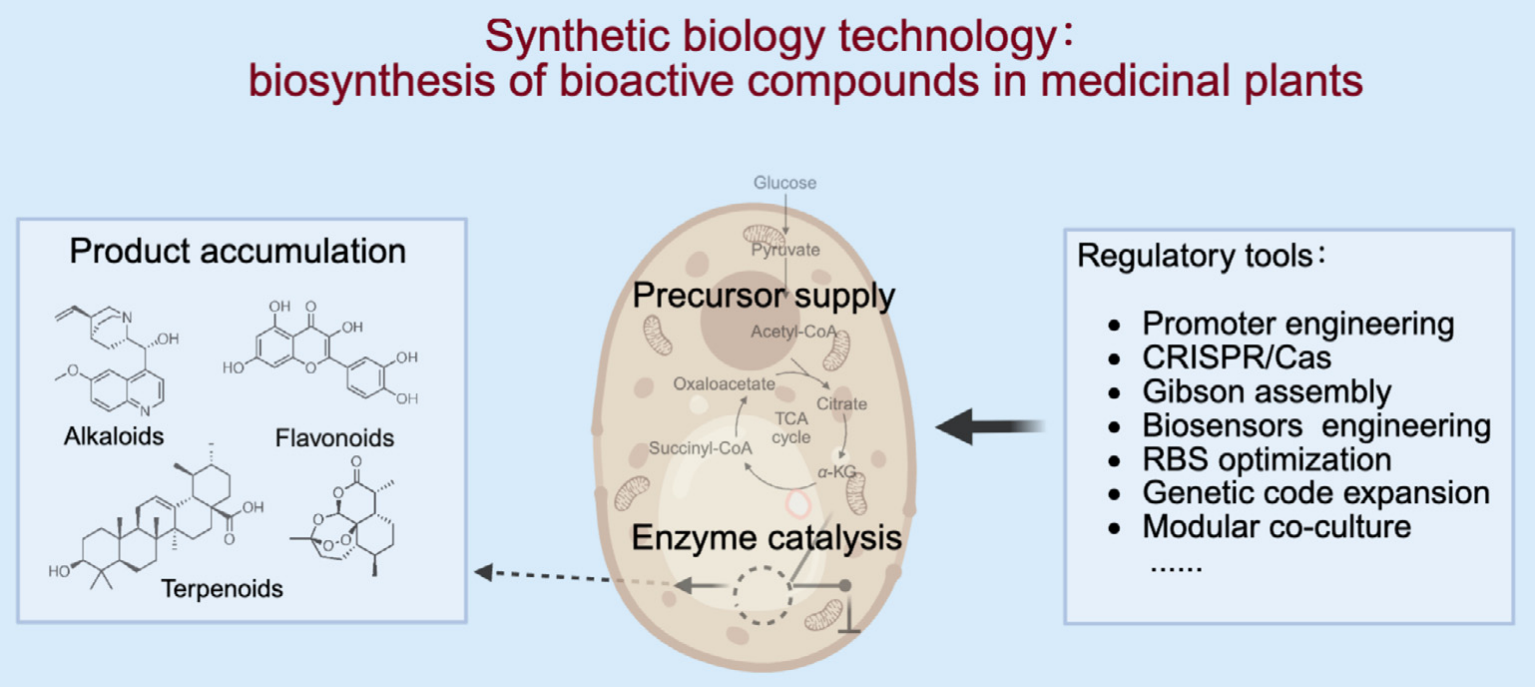
Schematic illustration of synthetic biology technology in the biosynthesis of bioactive compounds from medicinal plants.

However, synthetic biology faces several challenges in the biomanufacturing of bioactive compounds. The biosynthetic pathways of complex natural products, such as terpenoids, involve multi-enzyme cascades. The heterologous reconstruction of these pathways often results in suboptimal yields of the target bioactive compounds due to metabolic flux imbalance in host cells, insufficient activity of rate-limiting enzymes, limited cofactor supply, and incompatibility between genetic elements and host cells. Furthermore, engineered cell factories are prone to genetic instability and the accumulation of metabolic stress during large-scale fermentation processes, significantly compromising their robustness and production continuity. Regulatory tools in synthetic biology hold promise for overcoming these challenges. This section first categorizes bioactive compounds from medicinal plants according to structural diversity. Subsequently, recent advancements are outlined in regulatory tools and their applications in the heterologous production of bioactive compounds (Fig. 2). The main goal of this paper is to lay a foundation for efficient, sustainable microbial production of bioactive compounds via synthetic biology technology.

## 2 Overview of classification and biosynthesis of bioactive compounds from medicinal plants

Bioactive compounds from medicinal plants constitute a diverse group of substances. Among these, flavonoids, terpenoids, and alkaloids stand out as the most extensively studied classes, exhibiting broad application potential. The study of the biosynthesis of these compounds has also received significant attention (Table 1). In this section provide a comprehensive overview of the structure, distribution, function, and biosynthesis of these typical compounds.

**Table 1:** Biosynthesis of some typical bioactive compounds from medicinal plants by microorganisms.

Subclass	Product	Strains	Tools	Substrate	Yield	References
Flavonoids	Naringenin	*Saccharomyces cerevisiae*	Biosensor	Glucose	2.05 g/L	Mao et al., 2023
		*S. cerevisiae*	Biosensor	Sucrose	2513 mg/L	Tong, Li, Zhou, Zhang, Xu, & Zhou, 2024
		*Yarrowia lipolytica*	—	Glucose	8.65 g/L	Ru et al., 2025
		*S. cerevisiae*	Biosensor	Glucose	47.3 mg/L	Liu et al., 2022
		*Y. lipolytica*	Biosensor	Glucose and xylose	715.3 mg/L	Wei, Zhang, Shang, Zhou, & Ye, 2020
		*Escherichia coli*	Biosensor	Glycerol	98.71 mg/L	Hwang et al., 2023
		*E. coli*	Biosensor	Glycerol	277.2 mg/L	Jiang et al., 2022
	Eriodictyol	*Y. lipolytica*	Promoter replacement	Glucose	6.8 g/L	Yue et al., 2024
		*S. cerevisiae*	Promoter engineering and modular co-culture system	Glucose	132.1 mg/L	Zhang, Wu, Huang, Liu, & Wang, 2024
		*Corynebacterium glutamicum*	—	Glucose	14.1 mg/L	Wu, Liu, Liu, Yuwen, Koffas, & Zha, 2022
	Apigenin	*Y. lipolytica* and *E. coli*	Modular co-culture system	Glucose	168 mg/L	Marsan et al., 2024
	Kaempferide	*E. coli*	Modular co-culture system	Glucose	116 mg/L	Qiu, Liu, Li, Qiao, & Zhao, 2022
	Icaritin	*S. cerevisiae*	Dynamic regulation based on promoters	Glucose	130 μg/L	An et al., 2023
		*S. cerevisiae* and *E. coli*	Modular co-culture system	Glucose	19.7 mg/L	Wang et al., 2021
		*Y. lipolytica*	—	Glucose	247.02 mg/L	Sun et al., 2025
	Taxifolin	*Y. lipolytica*	—	Glucose	4.2 g/L	He et al., 2025
	Taxifolin	*S. cerevisiae*	—	Glucose	336.8 mg/L	Yang et al., 2020
	Taxifolin	*Y. lipolytica*	Genome editing	Glucose	50 mg/L	Zhang et al., 2025a
	Daidzein	*S. cerevisiae*	Dynamic regulation based on promoters	Glucose	85.4 mg/L	Liu, Liu, Li, Savolainen, Chen, & Nielsen, 2021
	Genistein	*S. cerevisiae*	—	Glucose	23.33 mg/L	Wang et al., 2024
	3′-Hydroxygenistein	*S. cerevisiae*	Promoter engineering	Glucose	1.40 mg/L	Tan et al., 2025
	Kaempferol, quercetin	*S. cerevisiae*	Genome editing	Glucose	956, 930 mg/L	Tartik, Liu, Mohedano, Mao, & Chen, 2023
Terpenoids	Crocetin	*Y. lipolytica*	Promoter engineering	Glucose	30.17 mg/L	Zhou, Park, Fu, Hapeta, Klemm, & Ledesma-Amaro, 2025
	Amorphadiene	*Y. lipolytica*	Modular co-culture system	Glucose	65.094 mg/L	Marsafar, Azi, Dou, & Xu, 2022
	Limonene	*S. cerevisiae*	Dynamic regulation	Glucose	2.63 g/L	Kong et al., 2023
	*β*-Elemene	*Ogataea polymorpha*	Dynamic regulation	Glucose	4.7 g/L	Ye, Gao, & Zhou, 2023
	Borneol	*S. cerevisiae*	Genome editing	Glucose	753 mg/L	Zhang et al., 2025b
	Geraniol	*Y. lipolytica*	Genome editing	Glucose	1 g/L	Agrawal, Yang, & Blenner, 2023
		*S. cerevisiae*	—	Glucose	9.5 g/L	Baker et al., 2025
	Licorice triterpenoid	*S. cerevisiae*	—	Glucose	4.92 g/L	Sun et al., 2024
	*β*-Carotene	*Y. lipolytica*	Genome editing	Glucose	3 968 mg/L	Gao et al., 2017
	Squalene	*Candida tropicalis*	Genome editing and dynamic regulation based on promoters	Glucose	433.06 mg/L	Wei et al., 2024
		*Y. lipolytica*	Genome editing	Glucose	35 g/L	Xu, Yang, Pan, Hua, Li, & Xu, 2024
	*β*-Farnesene	*O. polymorpha*	Genome editing	Methanol or glucose	14.7 g/L	Li et al., 2024
Alkaloids	Catharanthine	*Pichia pastoris*	Genome editing and promoter replacement	Methanol	2.57 mg/L	Gao et al., 2023
	Catharanthine	*P. pastoris*	Genome editing and promoter replacement	Methanol	2.57 mg/L	Gao et al., 2023
	Sanguinarine	*S. cerevisiae*	Temperature-responsive gene expression system	Glucose	448.64 mg/L	Gou et al., 2024
	Berberine	*S. cerevisiae*	Genome editing	Glucose	16.9 mg/L	Jiao et al., 2024
	Serpentine	*S. cerevisiae*	Genome editing	Glucose	8.85 mg/L	Bradley et al., 2023
	Chelerythrine	*S. cerevisiae*	Genome editing	Glucose	12.61 mg/L	Zhu et al., 2024
	Reticuline	*S. cerevisiae*	Genome editing	Glucose	425.2 mg/L	Jiao et al., 2024
	Reticuline	*Y. lipolytica*	Genome editing	Glucose	306 μg/L	Zhang et al., 2025a
	Norcoclaurine	*Y. lipolytica*	Genome editing techniques	Glucose	56 μg/L	Zhang et al., 2025a

### 2.1 Flavonoids

#### 2.1.1 Structure, distribution, and function of flavonoids

**Fig. 3:**
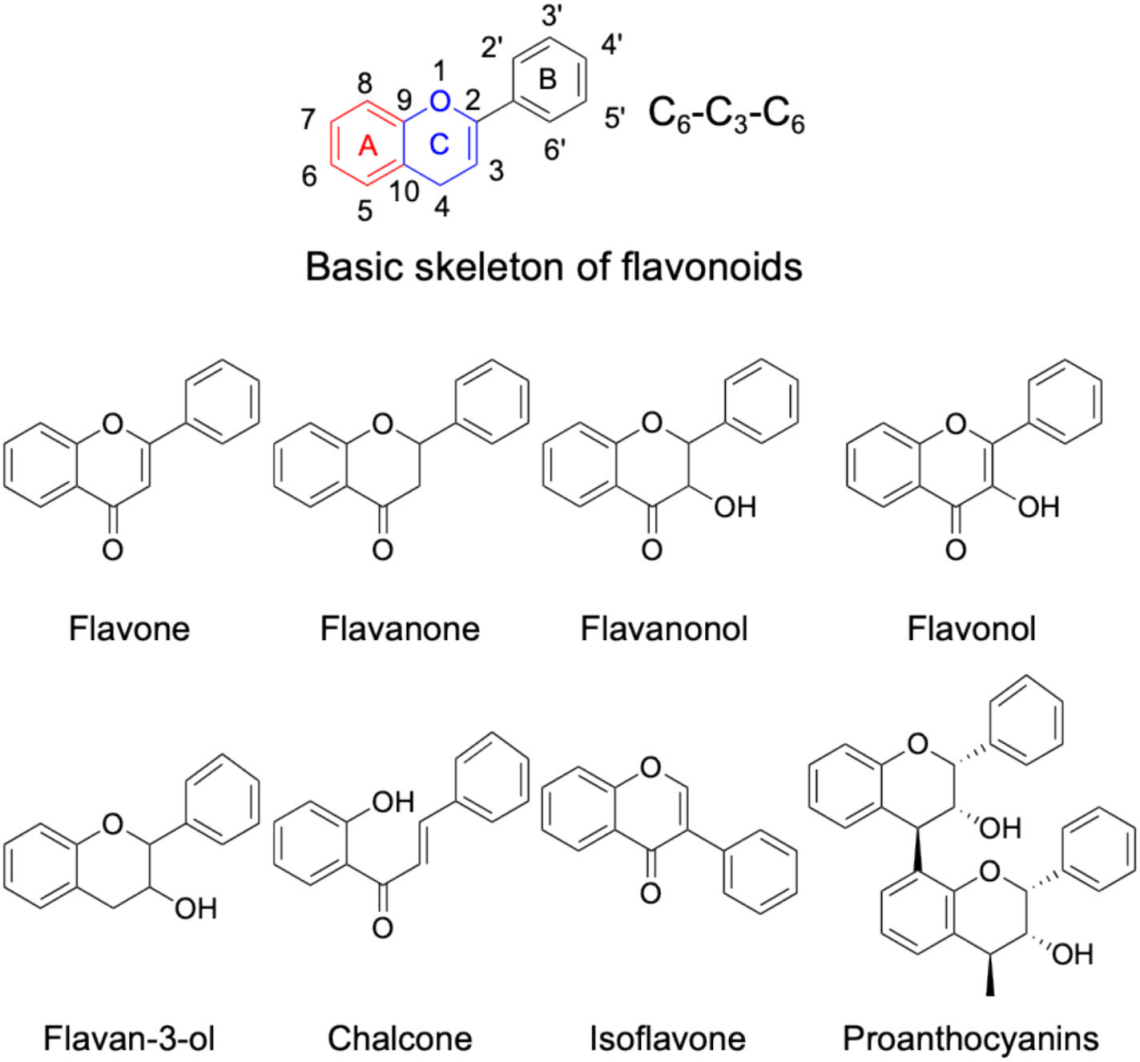
Framework structures of the primary classes of flavonoids.

Flavonoids are an important class of secondary metabolites in plants, and their structural feature is a diphenylpropane (C_6_-C_3_-C_6_) skeleton consisting of three rings (labeled A, B, and C in Fig. 3) (Shen, Wang, Gan, Liu, Wang, & Jin, 2022). Specifically, the differences in the levels of hydroxylation and oxidation of the C ring, together with changes in the substituents of the A and/or B rings, determine the unique structural classifications of flavonoids (Shamsudin et al., 2022). These structural modifications lead to diverse subclasses, including flavones, flavanones, flavonols, flavanonols, flavan-3-ols, chalcones, isoflavones, and proanthocyanins (Fig. 3). These compounds are crucial for plant defense mechanisms, as they help plants resist microbial infections and tolerate abiotic stresses (Zhuang et al., 2023). Meanwhile, flavonoids can also offer various health benefits to humans because of their biological activities, including antioxidant, anti-inflammatory, anticancer, antimicrobial, antiviral, and anti-cardiovascular properties (Liu, Yang, Guo, Jiang, Zhu, & Yang, 2025). Moreover, flavonoids profoundly impact plant physiology by modulating processes like phototropism, pollen tube growth, and responses to environmental stressors, including UV radiation and microbial infections (Yang, Yang, Zhao, Liu, & Jiang, 2016). Beyond their biological functions in plants, flavonoids are also widely present in tea, soybeans, citrus fruits, nuts, cereals, and berries, and serve as an indispensable component of the human diet (Zhong et al., 2023). It is worth noting that research interest in flavonoids with antibacterial activity within the realm of food safety is progressively increasing. In conclusion, as research continues to advance, flavonoids will assume even greater significance and functionality.

#### 2.1.2 Biosynthetic pathways of flavonoids

**Fig. 4:**
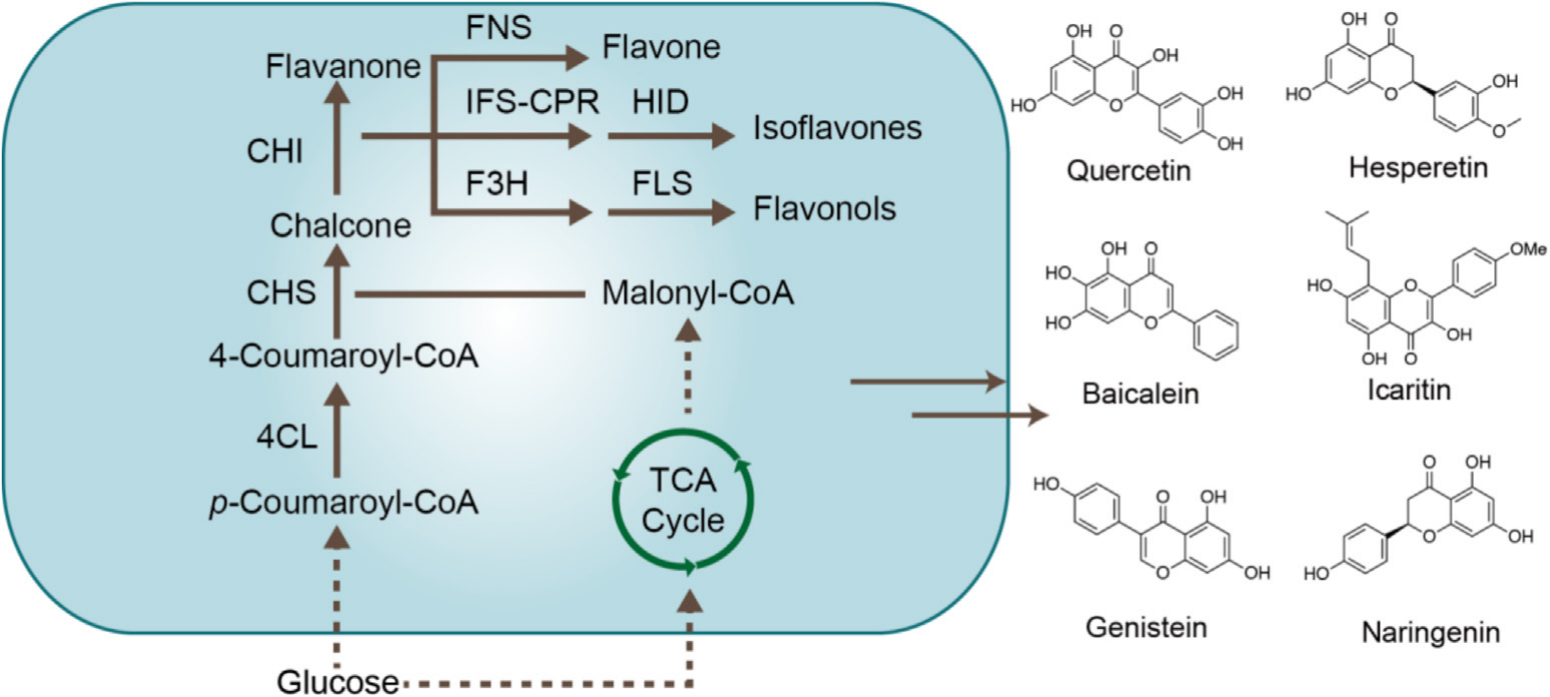
Biosynthetic pathway of flavonoids. TCA-tricarboxylic acid.

Currently, the biosynthetic pathways of flavonoids have been extensively studied and modified. This metabolic pathway can be categorized into four primary module (Fig. 4): Aromatic amino acid (precursor 1) biosynthesis module: This section focuses on the production of *L*-tyrosine and *L*-phenylalanine, which act as precursors for flavonoid formation. These amino acids are synthesized via the native shikimate pathway, where phosphoenolpyruvate (PEP) and erythrose-4-phosphate (E4P) are converted into prephenate (PPA), eventually leading to the generation of *L*-tyrosine or *L*-phenylalanine. Malonyl-CoA (precursor 2) biosynthesis module: Malonyl-CoA, a key biosynthetic precursor for flavonoid synthesis, is generated from acetyl-CoA through the catalytic action of acetyl-CoA carboxylase. Acetyl-CoA itself originates from multiple pathways, such as the citrate cycle, pyruvate dehydrogenase (PDH) complex, pyruvate bypass, and *β*-oxidation processes. (2*S*)-Naringenin biosynthesis module: One molecule of *p*-coumaroyl-CoA (derived from aromatic amino acids) and three molecules of malonyl-CoA undergo Claisen cyclization and dehydration reactions mediated by chalcone synthase (CHS) and chalcone isomerase (CHI), resulting in the formation of naringenin. It is worth noting that it is an intermediate for the synthesis of different subclasses of various flavonoids. Naringenin derived module: Following the formation of the flavanone backbone, the biosynthetic pathway branches out into multiple pathways, each leading to the production of distinct flavonoid subclasses. Isoflavones are synthesized via isoflavone synthase (IFS), while flavones are produced by flavone synthase (FNS). Flavanols are generated from the oxidation of flavanones catalyzed by flavanone 3-dioxygenase (F3H). Additional structural modifications, mediated by enzymes such as flavonol synthase (FLS), dihydroflavonol reductase (DFR), and anthocyanidin synthase (ANS), along with methyltransferases and prenyltransferases, result in the formation of a diverse array of structurally complex flavonoids, including flavonols, anthocyanins, and proanthocyanidins (Chen et al., 2019).

In conclusion, through the examination and modification of each of these components, it is possible to customize the flavonoid biosynthesis pathway. This customization can promote the production of a wide variety of flavonoid compounds that possess potential significance in industrial applications.

#### 2.1.3 De novo synthesis of flavonoids using cell factories

Recent advances in synthetic biology have facilitated the production of flavonoids through microbial cell factories, providing a sustainable and scalable alternative compared to traditional plant extraction. For example, icaritin, the primary active constituent of the traditional Chinese herb *Epimedii Folium* and a critical component in the liver cancer drug. Acolaritin, has been successfully synthesized from glucose in *E. coli* (19.7 mg/L) (Wang et al., 2021). In parallel, icariin, another anticancer flavonoid, was synthesized in *S. cerevisiae* (130 μg/L) (An et al., 2023). These breakthroughs highlight the potential of synthetic biology to produce complex compounds originating from plants, which hold considerable therapeutic value.

Besides these bioactive flavonoids, the production of critical intermediates like (2*S*)-naringenin has garnered significant attention. A study reported a yield of 588 mg/L in a 5-L fermentation system using *E. coli* (Zhou, Hao, & Zhou, 2020). Similarly, another study obtained a yield of 1.1 g/L by employing *S. cerevisia* (Zhang, Yu, Lyu, Zeng, & Zhou, 2021). Moreover, *Y. lipolytica* has also been engineered to produce 12.4 g/L of (2*S*)-naringenin in a 1-L fermentation system (Sáez-Sáe, Wang, Marella, Sudarsan, Cernuda Pastor, & Borodina, 2020). Beyond canonical flavonoids, other compounds have also been successfully synthesized through whole-cell catalysis. Research demonstrated the synthesis of baicalein from *L*-phenylalanine as the substrate using *E. coli*, achieving a yield of 271.6 mg/L (Ji, Li, Xu, Ren, & Wang, 2021). Similarly, studies synthesized kaempferol from *p*-coumaric acid as the precursor in a 1.5-L bioreactor using *S. cerevisiae* (66.3 mg/L) (Duan et al., 2017). Further expanding host diversity, research optimized taxifolin biosynthesis from glucose by reprogramming *Y. lipolytica*, yielding 134 mg/L in a 3-L fermentation system (Lv, Edwards, Zhou, & Xu, 2019). However, due to excessively long biosynthetic pathways and insufficient catalytic efficiency of key enzymes, the production titers of these compounds remain suboptimal, necessitating further optimization (Table 1). All in all, these findings highlight the versatility and efficiency of microbial cell factories in producing structurally diverse flavonoids.

### 2.2 Terpenoids

#### 2.2.1 Structure, distribution, and function of terpenoids

Terpenoids, also known as isoprenoids, constitute one of the most structurally diverse classes of natural products, with over 80 000 identified compounds to date. These compounds are defined by their molecular formula (C_5_H_8_)*_n_*, where ‘*n*’ represents the number of isoprene units. Based on the number of isoprene units and their structural organization, terpenoids are classified into several major groups: Hemiterpenes (C_5_), monoterpenes (C_10_), sesquiterpenes (C_15_), diterpenes (C_20_), sesterterpenes (C_25_), triterpenes (C_30_), tetraterpenes (C_40_), and polyterpenes (C_>40_) (Câmara et al., 2024). Representative examples of terpenoids include geraniol and menthol (monoterpenes), artemisinin and farnesol (sesquiterpenes), paclitaxel and triptolide (diterpenes), ursolic acid and oleanolic acid (triterpenes), and lutein and carotene (tetraterpenes) (Bergman & Dudareva, 2024).

Terpenoids are believed to have originated early in cellular evolution, playing a crucial role in the formation of primitive membranes with early precursors, such as diphytane glycerol ethers and polyprenols (Zwenger & Basu, 2008). They have since evolved into a highly diverse group found across a wide range of organisms, particularly in plants. Terpenoids are vital for plant growth, defense against pathogens and herbivores, resistance to stresses, and interactions with other organisms (Cheng, Lou, Mao, Lu, Wang, & Chen, 2007; Yang et al., 2020). Many terpenoids exhibit potent toxicity, serving as chemical defenses and contributing significantly to their medicinal and economic value. They find applications in industries such as fragrances, pharmaceuticals, biofuels, and natural rubber (Bergman, Davis, & Phillips, 2019). For instance, monoterpenes with anticancer activity, including perilla alcohol and geraniol, sesquiterpenes like artemisinin and costunolide, diterpenes like paclitaxel, and triterpenes like betulinic acid and cucurbitacins have been developed into drugs. The volatile monoterpene menthol is commonly used in fragrances, cosmetics, and flavorings, while pyrethroids and limonoid compounds serve as insecticides, and bisabolene, limonene, and pinene are used in biofuels (Huang et al., 2023).

#### 2.2.2 Biosynthetic pathways of terpenoids

**Fig. 5:**
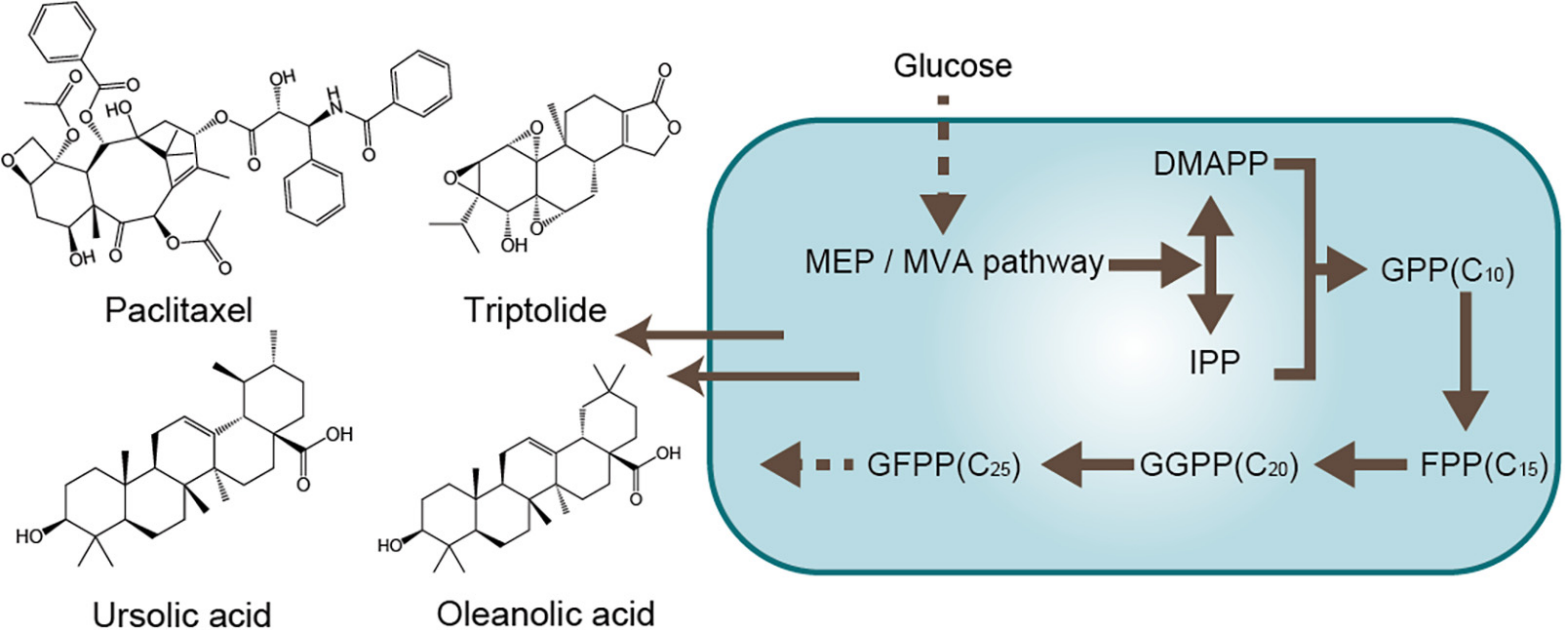
Biosynthetic pathway of terpenoids. FPP-farnesyl diphosphate, GGPP-geranylgeranyl diphosphate, GFPP-geranylfarnesyl diphosphate.

The biosynthetic pathway of terpenoids can be broadly categorized into three main segments: The upstream metabolic pathway, which primarily involves the conversion of carbon sources (e.g., glucose and xylose) into precursor molecules for terpene synthesis, including pyruvic acid, glyceraldehyde-3-phosphate, and acetyl-CoA, along with cofactors and ATP via central carbon metabolism, the midstream metabolic pathway, specifically the synthesis pathways of the C_5_ skeleton isoprene diphosphate (IPP) and dimethylallyl diphosphate (DMAPP) (Kuzuyama & Seto, 2012), and the downstream terpene synthesis pathway, which involves the assembly of terpene skeletons and their subsequent modifications by terpene synthases, leading to a diverse array of functional terpene compounds (Fig. 5) (Volke, Rohwer, Fischer, & Jennewein, 2019). The midstream metabolic pathway is uniquely bifurcated into two distinct routes: The 2-*C*-methyl-*D*-erythritol-4-phosphate (MEP) pathway and the mevalonate (MVA) pathway, each distinguished by their specific sources of IPP and DMAPP. Notably, the MEP pathway utilizes pyruvate and glyceraldehyde 3-phosphate as precursors and is predominantly located in plant plastids, cyanobacteria, and most bacteria. Conversely, the MVA pathway utilizes acetyl-CoA as a precursor and is chiefly found in archaea, specific bacteria, plant cytoplasm, and the majority of eukaryotes (Di et al., 2023). Notably, some archaea possess an alternative form of the MVA pathway that can bypasses mevalonate pyrophosphate to generate IPP. To address the challenges posed by the long natural terpenoid synthesis pathways (MEP and MVA) and the complexities of metabolic regulation, scientists have engineered artificial terpenoid synthesis pathways. An example of such a pathway is the isopreneol utilization pathway (IUP), which synthesizes IPP in just two steps using isopreneol as a substrate (Zhang et al., 2023).

#### 2.2.3 De novo synthesis of terpenoids using cell factories

Numerous studies have demonstrated the successful biosynthesis of terpenoid compounds in microbial cell factories, highlighting the potential of these systems for producing high-value bioactive molecules. For example, quillaja saponin-21 (QS-21), a highly effective vaccine adjuvant approved for use in multiple vaccines, was synthesized by the Jay Keasling research group. The team fine-tuned the metabolic flux and optimized the expression and balance of 38 heterologous enzymes, ultimately producing QS-21 analogs through a biosynthetic platform (Liu et al., 2024). Additionally, the same research group engineered *S. cerevisia* to produce artemisinin, the precursor to the anti-malarial drug artemisinin. This breakthrough not only stabilized the global supply of artemisinin, but also made it more accessible and affordable, particularly in malaria-endemic regions (Paddon et al., 2013).

Beyond terpenoids with significant medicinal value, various terpenoids with versatile funticons are also being widely synthesized. For instance, research investigations examined 122 sesquiterpene scaffolds, identifying nine high-yielding strains and assessing their potential as high-energy aviation fuels (Huang et al., 2023). Similarly, studies achieved concentrations of 966.55 mg/L nerol and 87.20 mg/L borneol in *E. coli* (Lei, Qiu, Wu, Qiao, Qiao, & Zhao, 2021). Additionally, research reported the successful synthesis of glycyrrhizin (GL) and glycyrrhetic acid 3-*O*-mono-*β*-*D*-glucuronide (GAMG) in *S. cerevisiae*, yielding production levels of 5.98 mg/L and 2.31 mg/L for GL and GAMG, respectively (Xu et al., 2021). These studies underscore the growing potential of microbial cell factories to efficiently produce terpenoid compounds for diverse applications, including drug development, industrial materials, and sustainable energy solutions. However, their output remains relatively low and requires further enhancement (Table 1).

### 2.3 Alkaloids

#### 2.3.1 Structure, distribution, and function of alkaloids

Alkaloids, a class of nitrogen-containing organic compounds that occur naturally and are typically derived from amino acids, exhibit complex and diverse molecular structures. These structures primarily consist of carbon, hydrogen, and nitrogen, with the inclusion of oxygen, sulfur, chlorine, bromine, and phosphorus (Bui, Rodríguez-López, & Dang, 2023). The structural characteristics of alkaloids, including cyclic structures and a variety of functional groups, significantly influence their biological activity and pharmacological effects. Alkaloids can be categorized based on their chemical structure and nitrogen source into several types, such as indole (e.g., colchicine), pyridine (e.g., nicotine), tropane (e.g., atropine), and isoquinoline (e.g., morphine) (Schläger & Dräger, 2016).

Alkaloids are widely distributed in plants, animals, bacteria, and fungi, particularly as secondary metabolites in plants. They play critical roles in plant defense mechanisms against biotic stressors (e.g., pathogens and herbivores) and in attracting pollinators to promote reproduction (Jha & Mohamed, 2022). Currently, approximately 10 000 alkaloids have been identified, with an estimated 100 new compounds discovered annually. Therein, terpenoid indole alkaloids (TIAs) constitute the largest class and are primarily found in plant families like apocynaceae, magnoliaceae, and rubiaceae (Taha, 2003). For humans, alkaloids exhibit significant pharmacological activities and are widely utilized in drug development (Mishra, Tripathi, Kishore, Singh, Singh, & Tiwari, 2017). They have diverse therapeutic effects, including anticancer, antibacterial, analgesic, and antidepressant properties (Ng, Or, & Ip, 2015). Notable examples include morphine and codeine, which are used for pain relief and anesthesia, and nicotine, which has stimulating effects in tobacco products. The remarkable diversity and potent biological activities of alkaloids make them indispensable components of medicinal plants, offering abundant resources and broad application prospects for the development of drugs and natural products (Debnath et al., 2018).

#### 2.3.2 Biosynthetic pathways of alkaloids

**Fig. 6:**
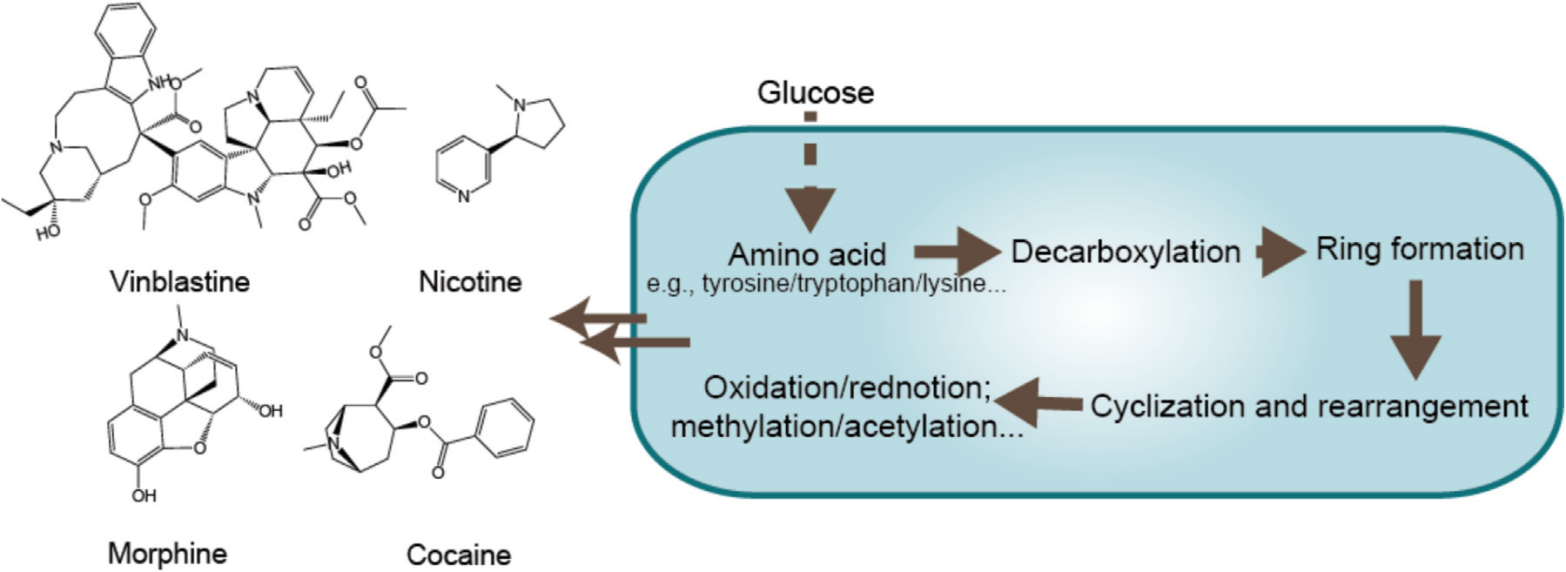
Biosynthetic pathway of alkaloids.

Alkaloids represent a structurally diverse group of compounds whose biosynthetic pathways exhibit shared characteristics. These pathways generally initiate with amino acid precursors and most alkaloids are derived from amino acids such as ornithine, lysine, phenylalanine, tyrosine, tryptophan, and histidine. The initial step usually entails the decarboxylation of these amino acids, a process catalyzed by decarboxylases and facilitated by the cofactor pyridoxal phosphate (PLP). In many alkaloid biosynthesis pathways, the amino group of the amino acid reacts with the aldehyde group of PLP to form an imine intermediate. This intermediate subsequently undergoes decarboxylation, generating a carbanion that is protonated and hydrolyzed to produce the corresponding amine, thereby regenerating PLP for subsequent reactions (Fig. 6) (Facchini & De Luca, 2008).

Monoterpenoid indole alkaloids, such as vinblastine, are bioactive compounds from plants with significant biological activity. (Yu & Yong, 2021). Herein, this study takes it as a representative example for introduction Strictosidine, a key skeletal compound in the biosynthesis of monoterpene indole alkaloids, is produced by the fusion of tryptamine and secologanin (Zhu et al., 2015). This process, known as the upstream synthesis pathway, primarily occurs via two pathways: The iridoid pathway and the indole pathway. Under the action of strictosidine synthase (STR), strictosidine is formed by the condensation of tryptamine and secologanin. The downstream synthesis pathway involves the generation of various monoterpenoid indole alkaloids, starting from strictosidine as the initial precursor and facilitated by diverse enzymes (Yamazaki et al., 2003, Vadhel et al., 2023). Well-studied biosynthetic pathways involve those of vinblastine, tabersonine, vincristine, heteroyohimbine and their derivatives from *Catharanthus roseus* and *Vinca minor* L., as well as ajmaline from *Rauvolfia verticillata* (Lour.) Baill. (Yu & Yong, 2021).

#### 2.3.3 De novo synthesis of alkaloids using cell factories

Advancements in synthetic biology have enabled the *de novo* synthesis of alkaloids through engineered microorganisms. A landmark achievement was made by Christina D. Smolke’s research group, which successfully achieved the first artificial biosynthesis of tropane alkaloids in *S. cerevisiae*. They designed a biosynthetic pathway involving five functional modules to produce compounds like hyoscyamine and scopolamine from simple sugars and amino acids. This process involved identifying missing enzymes, engineering proteins for functional expression, and optimizing fermentation conditions, ultimately resulting in a yield of 60 mg/L (Srinivasan & Smolke, 2020). Similarly, research by the Keasling group engineered *S. cerevisiae* to produce monoterpenoid indole alkaloids (MIAs) and their halogenated derivatives. They also made significant progress in producing vinblastine precursors and other complex drugs by introducing and fine-tuning numerous genes in yeast (Bradley et al., 2023, Zhang et al., 2022). To address temporal regulation challenges, Research developed a temperature-responsive gene expression system based on intein-mediated control (SIMTeGES), enabling the complete biosynthesis of sanguinarine and its halogenated derivatives in yeast (Gou et al., 2024). Concurrently, Studies expanded the alkaloid biosynthetic toolkit by characterizing NascB, a promiscuous P450 enzyme enabling efficient pyrroloindoline scaffold formation (Tian et al., 2018). Nevertheless, because of the overly extended biosynthetic pathways and the inadequate catalytic performance of critical enzymes, the yield of alkaloids is still less than ideal, thus requiring additional improvements (Table 1). Collectively, these studies contribute to the establishment of microbial platforms for sustainable production of essential alkaloid compounds from medicinal plants.

### 2.4 Other compounds

In addition to flavonoids, terpenes, and alkaloids, synthetic biology and metabolic engineering have enabled the biosynthesis of diverse bioactive compounds, including glycosides (Guan et al., 2025), polyketides (Gayen et al., 2020), non-ribosomal peptides (Chen et al., 2023), saccharides (Rana & Upadhyay, 2020), ribosomally synthesized peptides (Liu, Yang, Guo, Jiang, & Sun, 2024), and post-translationally modified peptides (Wang et al., 2022). These compounds hold significant potential for applications in medicine, agriculture, and food science. Recent advances in microbial biosynthesis have achieved impressive yields through innovative strategies, such as the selection of optimal microbial chassis (e.g., *E. coli*, *S. cerevisiae*, and *Y. lipolytica*) and the optimization of pathways, synthetic promoters, and enzyme co-expression. Consequently, these developments have led to more robust microbial strains and cost-effective fermentation processes, enabling large-scale production of medicinal plant-derived compounds. In the following sections, we will explore the cutting-edge tools and strategies driving this field, which are shaping the future of biotechnological innovation.

## 3 Development of synthetic biology regulatory tools and their applications

**Fig. 7:**
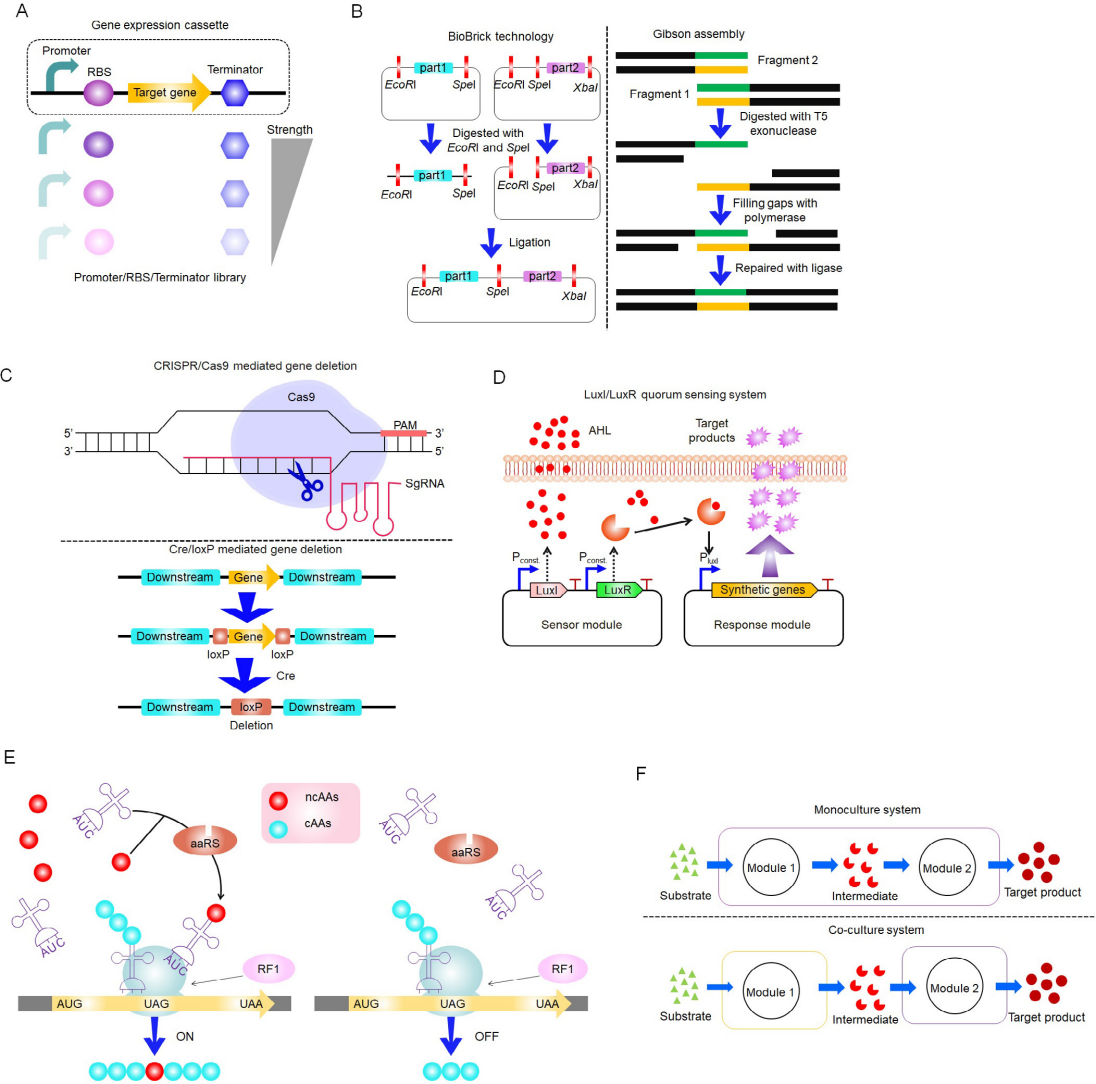
Synthetic biology regulatory tools. (A) Gene expression regulatory parts, including promoter, ribosome binding sites (RBS), and terminator. (B) The illustrative figure for BroBrick technology and Gibson assembly. (C) Scheme of the clustered regularly interspaced short palindromic repeats (CRISPR)/CRISPR-associated protein 9 (Cas9) system and the causes recombination (Cre)/locus of X-over P1 (loxP) system for genome editing. (D) The *N*-acyl homoserine lactone synthase (LuxI)/transcriptional regulator (LuxR) quorum sensing system in *Vibrio fischeri*. LuxI is the signal molecule AHL synthetase. LuxI serves as the enzyme responsible for synthesizing AHL signal molecules. When LuxR binds to AHL, it triggers the activation of gene transcription downstream of the P_LuxI_ promoter. (E) The schematic diagram for genetic code expansion technology. (F) Collaborative interactions within co-culture systems enhance the yield of the desired product.

Microbial cell factories possess the remarkable ability to convert inexpensive biomass feedstocks into high-value compounds, serving as a cornerstone platform for biomanufacturing. However, several challenges hinder the construction of efficient microbial cell factories using synthetic biology, including overly long metabolic pathways, insufficient catalytic efficiency of key enzymes in the pathway, and incompatibility between genetic elements and host cells, all of which result in low yields of target bioactive compounds. The development and application of synthetic biology regulatory tools offer significant potential by enabling precise control over microbial metabolism. In this section introduces some regulatory tools in synthetic biology that enhance the biosynthesis of bioactive compounds (Fig. 7). First, foundational tools such as gene expression regulatory parts and DNA assembly technique enable modular construction of gene expression cassette. Second, genome editing technologies and biosensor-based dynamic regulation systems allow real-time pathway optimization through host genome modification and feedback-controlled expression. Third, emerging strategies including genetic code expansion and modular co-culture systems address challenges in pathway compartmentalization and metabolic burden distribution. Collectively, these multi-level interventions demonstrate how synthetic biology tools overcome intrinsic limitations of microbial cell factories, highlighting their critical role in synthesizing pharmaceutically bioactive compounds and advancing biomanufacturing applications.

### 3.1 Gene expression regulatory parts

Achieving precise control over gene expression stands as one of the primary challenges in synthetic biology, particularly when aiming to optimize metabolic pathways (Yang et al., 2024). To tackle this issue, it is crucial to conduct a systematic evaluation of the functional attributes of gene expression components. For example, promoters modulate the level of gene expression by governing the rate of transcription initiation, ribosomal binding sites (RBS) affect translational efficiency, and terminators ensure the precision of transcriptional termination (De Nijs et al., 2020, Ito et al., 2020, Zhang et al., 2022, Xiao et al., 2024). Notably, for model microorganisms such as *E. coli*, *Bacillus subtilis*, and *S. cerevisiae*, scientists have established multi-dimensional databases. These databases quantify the expression strength and dynamic range of various elements, offering modular resources for metabolic engineering applications (Fig. 7A). Building on this framework, the design of metabolic pathways frequently entails integrating multiple gene expression modules. For instance, coupling promoters with RBS can harmonize the expression levels of enzymes that limit reaction rates, while weaker terminators can reduce the impact of transcriptional read-through on host metabolism (Chen et al., 2019, Lee et al., 2024, Liu et al., 2022). Collectively, these gene expression elements serve as the foundation for developing highly efficient microbial cell factories.

The control of transcription is the primary regulatory step in bacterial gene expression (Jensen & Galburt, 2021). Therein, the promoter serves as a critical transcriptional signal that precisely regulates target gene expression at the transcriptional level by modulating RNA polymerase recruitment efficiency (Brázda et al., 2021, Cazier and Blazeck, 2021). Leveraging this characteristic, promoter engineering has been widely applied to optimize biosynthetic pathways. For example, studies demonstrated that eriocitrin production was enhanced eriocitrin production 5-fold by pairing promoters of varying intensities to balance metabolic fluxes between glycosylation reactions and glycosylated donor synthesis (Xiao et al., 2023). Meanwhile, research expanded the promoter toolkit for *O. polymorpha* by integrating transcriptomic data, constructing a library comprising 13 constitutive, two growth-stage-dependent, and two heterozygous promoters. Using this strategy, the engineered strain YY150U achieved a *β*-elemene yield of 5.24 g/L during shake flask-fed batch fermentation (Ye et al., 2024). In addition, by manipulating the inducible promoter to alleviate product toxicity and optimize the expression level of geraniol synthase, the production of geraniol was successfully increased to 1.68 mg/L (Jiang et al., 2017). These examples illustrate how promoter engineering can enhance target product synthesis through mechanisms such as coordinating carbon flux distribution and regulating rate-limiting enzyme catalytic efficiency. However, the existing studies continue to encounter challenges, including inadequate characterization of endogenous promoters, a limited dynamic range for regulation, and insufficient host adaptability. In particular, the trade-off between component orthogonality and the global allocation of metabolic fluxes within synthetic pathways remains unresolved. Consequently, the development of synthetic promoters with an extended dynamic range and enhanced host compatibility will represent a critical direction for advancing the industrial application of this technology.

Terminators are also one of the crucial regulatory elements in gene expression (Ray-Soni et al., 2016). Terminators can influence mRNA stability and abundance, rendering them essential for the completion of the transcription process (Curran et al., 2013, Yamanishi et al., 2013). Given these characteristics, terminators have been extensively utilized in metabolic engineering and synthetic biology. For instance, research achieved a 35% and 34% increase in the production of *α*-amylase and poly-*γ*-glutamic acid by employing an artificially designed short terminator T1 (Rao et al., 2022). To identify termination sequences suitable for regulating protein expression levels in *P. pastoris*, research developed a ‘terminator catalog’ through the evaluation of 72 sequences that exhibited a regulatory range of 17-fold (Ito et al., 2020). Furthermore, research into synthetic terminators have also been conducted. Study designed and constructed a series of components ranging from 38 to 75 bp as minimal terminators, with the most effective variants enhancing fluorescence intensity by up to 3.7 times (Curran et al., 2015). Despite current limitations in this field, its potential within synthetic biology has garnered considerable interest from researchers. Future studies are expected to further elucidate the role of terminators in regulating gene expression within microbial cells.

In addition to promoters and terminators, ribosome binding sites (RBS) play a crucial role in translation level regulation (Rao et al., 2024). They directly influence both the abundance and quality of proteins by affecting translation efficiency and fidelity (Faure et al., 2016). Consequently, RBS libraries with varying strengths serve as valuable tools for gene expression regulation, thus alleviating the problem of insufficient catalytic efficiency of key enzymes (Mutalik et al., 2013). For instance, studies optimized the ribosome binding site of sesquiterpene synthase, resulting in the optimal strain R10 producing 151.25 mg/L of *β*-elemene (Fordjour et al., 2023). Similarly, research engineered the strain CgSq27p to produce 15.15 mg/L *α*-amyrin by modulating the expression level of the key enzyme MdaASCgco using RBS sequences with varying strengths (Li et al., 2025). These studies not only highlight the significant potential of RBS optimization in metabolic engineering but also offer valuable insights for analogous research endeavors. Overall, as a central component of translational regulation, RBS plays a crucial role in optimizing gene expression. By precisely tuning the RBS sequence, both the expression level and functional characteristics of the target protein can be enhanced, thereby providing robust support for advancements in synthetic biology and metabolic engineering. Looking ahead, with the continued development of high-throughput screening technologies and computational prediction models, RBS optimization strategies are anticipated to become increasingly precise and efficient, further driving innovation in related fields.

### 3.2 DNA assembly techniques

DNA assembly is a crucial fundamental technology in synthetic biology and the cornerstone of gene expression cassette and pathway engineering (He et al., 2023; Shao et al., 2009). Beginning in the 1970 s, this area has undergone substantial transformations, initially through restriction endonuclease-based digestion and ligation techniques, which established the groundwork for precise DNA manipulation. In response to limitations concerning scalability and adaptability, scientists have invested considerable effort into creating more efficient, accurate, modular, and economical DNA assembly technologies (Gibson et al., 2009). Nevertheless, challenges persist in harmonizing assembly processes across various host organisms. Ongoing refinement of these methods, particularly via automation and standardization, remains essential to satisfy the increasing requirements of synthetic biology applications.

Depending on the distinct assembly mechanisms, DNA assembly methods can typically be classified into several categories: Those that rely on DNA polymerases, endonucleases (e.g., BioBrick, BglBrick, ePathBrick, MASTER, Golden Gate), exonucleases [e.g., Gibson, Sequence and Ligation Independent Cloning (SLIC)], and enzyme-independent assembly strategies (e.g., enzyme-free cloning, thermal paste annealing) (Fig. 7B) (Bird et al., 2022, García-Nafría et al., 2016, Li and Elledge, 2007, Liang et al., 2017, TerMaat et al., 2009). A notable study introduced a seamless and highly efficient start-stop assembly method for multi-element integration, enabling the incorporation of up to 60 elements into an expression plasmid with 15 expression units. Each unit comprises four key components: A promoter, an RBS sequence, a CDS, and a terminator. This technique greatly enhances the assembly of extremely long pathways and the optimization of expression unit combinations (Taylor, Mordaka, & Heap, 2019). Overall, these approaches offer a practical and effective means for building longer and more intricate genomic sequences.

Currently, DNA assembly methods play a crucial role in synthetic biology by enabling the construction and optimization of intricate metabolic pathways. research designed several modules incorporating genes from opioid biosynthetic pathways, successfully combining 21 diverse genes along with two yeast-originated genes through the Gibson and DNA assembler techniques. These modules were subsequently incorporated into yeast chromosomes or plasmids for expression, leading to engineered strains that could produce opioid compounds (Galanie, Thodey, Trenchard, Filsinger Interrante, & Smolke, 2015). Additionally, studies applied the Golden Gate approach to develop a library of violacein-producing strains in *Y. lipolytica*, attaining a violacein yield of 70.04 mg/L under optimized growth conditions (Tong, Zhou, Zhang, & Xu, 2021). In conclusion, DNA assembly technology offers a powerful and flexible framework for synthesizing bioactive compounds via extensive and complicated metabolic routes.

### 3.3 Genome editing techniques

The development of efficient genome editing tools is crucial for the construction and improvement of microbial cell factories (Lv et al., 2023, Qian et al., 2023). Therein, the CRISPR/Cas9 system has become a significant area of focus in recent studies (Lim & Lee, 2024). This innovative technology allows for highly precise modifications to target genes, enabling actions such as replacement, insertion, suppression, or removal of specific genes to achieve desired cellular characteristics (Westbrook et al., 2016, Pacesa et al., 2024). Consequently, the CRISPR/Cas9 system has been successfully applied across a range of microorganisms, including *E. coli, Bacillus subtilis*, *C. glutamicum*, *S. cerevisiae* and so on (Cai et al., 2022, Mózsik et al., 2021, Peng et al., 2017, Sachla et al., 2021). The core mechanism of the CRISPR/Cas9 system relies on the Cas9 protein (Mir et al., 2018). Guided by the RNA sequence, the Cas9 protein identifies DNA regions containing the protospacer adjacent motif (PAM) and induces accurate cuts at the designated location, leading to a double-strand break (Fig. 7C). Following the introduction of a break, the cell activates its intrinsic repair pathways, such as non-homologous end joining (NHEJ) or homologous recombination (HR), to restore the damaged site (Lv et al., 2023). Consequently, a variety of targeted mutations can be generated. Currently, To date, the CRISPR/Cas9 system has been effectively utilized for the biosynthesis of terpenes, flavonoids, and alkaloids in microorganisms (Marsafari and Xu, 2020, Moon et al., 2020, Sun et al., 2024). For example, a study employed the CRISPR/Cas9 technology to integrate expression units of four key enzymes involved in resveratrol biosynthesis into the genome of *S. cerevisiae*. After optimization, the resveratrol production was increased to 151.6 mg/L (Costa et al., 2021). Moreover, this approach has been extended to modify *E. coli* for the development of *L*-tyrosine-producing chassis cells. Further refinements enabled efficient (*S*)-canadine biosynthesis, achieving a final titer of 165.74 mg/L (Zhang, Wu, Huang, Liu, & Wang, 2024).

Despite its advantages, the CRISPR/Cas9 system encounters several challenges, such as sequence limitations, off-target effects, and potential genetic toxicity (Pacesa et al., 2022, Sreekanth et al., 2020). To overcome these obstacles, innovative techniques have been introduced to improve the efficiency and specificity of genome editing. For instance, in 2018, research devised a CRISPR-based gRNA-free one-step genome editing method called CAGO for use in *E. coli*. This technique bypasses the need for designing and constructing gRNAs by incorporating a universally optimal N20 sequence into the chromosome through homologous recombination. Subsequently, CRISPR/Cas9 generates double-strand breaks, initiating intrachromosomal recombination events that lead to the desired genomic modifications (Zhao et al., 2017). Meanwhile, studies developed a system referred to as gRNA-tRNA-array CRISPR/Cas9 (GTR-CRISPR), in which multiple guide RNAs are linked together and expressed through tRNA sequences. This approach greatly improves both the capability and effectiveness of gene editing in *S. cerevisiae*. By employing this system, plasmids can be designed to facilitate the simultaneous modification of up to eight genes within seven days of obtaining primers, achieving an editing efficiency of 87%. To date, this remains the most effective gene-editing system for *S. cerevisiae*, capable of targeting the highest number of genes concurrently (Zhang et al., 2019a). Furthermore, CRISPR interference technology can be applied to adjust the expression levels of target genes (Zhao et al., 2021). For example, the use of CRISPR interference successfully reduced the expression of lanosterol synthase in *S. cerevisiae*, leading to a remarkable 14.4-fold increase in protopanaxadiol (PPD) production at the shake flask level (Lim et al., 2021). Overall, the emergence of novel technologies provides a powerful and efficient foundation for synthetic biology research, further enhancing the CRISPR/Cas9 system. This not only lowers the technical hurdles for its application but also demonstrates substantial potential for optimizing intricate metabolic pathways.

Furthermore, site-specific recombinases serve as an additional effective genome editing tool (Zhang et al., 2019b). These recognition sites originate from the Cre recombinase/loxP (locus of X-over P1) site-specific recombination system of the P1 bacteriophage and the flippase (Flp)/Flp recognition target (FRT) recombination system in yeast (Pomerantsev et al., 2017). Both Cre and Flp belong to the tyrosine recombinase family, which identifies their respective 34 bp target sequences (loxP and FRT) and facilitates site-specific recombination (Meinke et al., 2016). This mechanism functions by integrating specific recognition sequences into both the genome and the target DNA. Upon expression of the corresponding recombinases, these sequences undergo recombination, enabling accurate insertion, deletion, or replacement of large DNA fragments (Fig. 7C) (Olorunniji et al., 2016). The Cre/loxP recombinant system has been widely applied in *S. cerevisiae* for precise gene deletion and integration, whether involving single or multiple genes. Research utilized the high recombination efficiency of the Cre/loxP system, combined with the high integration rate of 26S rDNA, to establish a flexible strategy for constructing multi-copy metabolic pathways in *Y. lipolytica*. The optimized strain achieved accumulations of 71.2 mg/L naringenin, 54.2 mg/L eriodyctiol, and 48.1 mg/L taxifolin (Lv, Marsafari, Koffas, Zhou, & Xu, 2019). Additionally, this system has been effectively employed for gene deletion and integration in *E. coli* and *B. subtilis* (Yu et al., 2002, Yan et al., 2008, Niu et al., 2024). For example, Studies demonstrated the Cre/loxP system to specifically eliminate an enzyme involved in quercetin degradation in *B. subtilis*, leading to an increased isoquercitrin production of 1.64 g/L (Niu et al., 2024). In conclusion, the implementation of efficient gene editing tools can substantially decrease the time required for constructing microbial cell factories while enhancing the biosynthesis of bioactive compounds from medicinal plants.

### 3.4 Dynamic synthetic biology regulatory tool based on biosensors

Achieving an equilibrium in carbon metabolism flux between cellular growth and the synthesis of target products poses significant challenges when relying on traditional static regulation methods (gene overexpression or knockout) (Taylor, Mordaka, & Heap, 2019). Conversely, dynamic regulatory systems have demonstrated their efficacy in maintaining this balance, resulting in the efficient production of bioactive compounds (Li et al., 2022). As a fundamental component of dynamic regulation systems, biosensors can convert biomolecules, cellular microbiota, and various environmental changes, such as temperature, light, and electrochemical signals, into gene regulatory signals that facilitate programmed cellular responses (Han et al., 2023). Biological systems employ diverse recognition elements with distinct response mechanisms, among which transcription factors (TFs) and riboswitches are the most widely characterized and applied (Chai et al., 2021). Research engineered a biosensor specific to *p*-coumaric acid and confirmed its functionality in both *E. coli* and *C. glutamicum*. This biosensor was subsequently employed to screen yeast cells, enhancing *p*-coumaric acid production (Siedler et al., 2017). In a similar vein, studies devised an efficient screening tool based on artificial naringenin riboswitches, which enabled rapid identification of mutant strains exhibiting enhanced CHS activity. The optimal strain was identified through two rounds of screening from the CHS mutant library, producing 55.46 mg/L naringin, which is 38% higher than the wild-type strain (Hwang, Milito, Yang, Jang, & Jung, 2023). Notably, these studies also reveal persistent limitations in constructed biosensors, including narrow operational ranges, constrained dynamic response capabilities, and suboptimal detection thresholds, which require systematic optimization through engineering strategies, such as promoter-RBS tuning or modular sensor component replacement.

Moreover, biosensors developed based on quorum sensing (QS) systems are considered a highly potential tool in the field of synthetic biology (Dinh & Prather, 2019). QS is an autoinduction system in microorganisms that dynamically regulates the expression levels of intracellular target genes during fermentation (Fig. 7D). This regulation is dependent on cell density, which allows it to function without external inducers and operate independently of metabolic pathways (Cui et al., 2019). For example, research constructed an inducer-free biosynthetic pathway for bisabolene production in *E. coli* using the LuxI/LuxR QS system. To enhance the regulatory efficiency of the QS system on bisabolene synthesis, constitutive promoters with different strengths were evaluated to control the expression levels of LuxI and LuxR. Ultimately, the yield of bisabolene reached 1.1 g/L, representing a 44% improvement over the induction system (Kim et al., 2017). Studies utilized the QS system to achieve a twofold increase in the titer of oxygenated taxanes (Glasscock et al., 2021). These results indicate that even with complex metabolic networks, it is still possible to regulate metabolic pathways by fine-tuning several critical influencing factors. Additionally, combining QS systems with other regulatory technologies can enable more precise control over metabolic networks while expanding their application range.

### 3.5 Genetic code expansion technique

In microbial biosynthesis of bioactive compounds, chassis cells require precise redirection of metabolic flux from biomass formation toward target product synthesis (Liu et al., 2017). However, excessive metabolic diversion may negatively affect cell growth and consequently reduce production efficiency (Nielsen & Keasling, 2016). Therefore, achieving an optimal balance between cell growth and product synthesis presents a critical challenge in constructing efficient microbial cell factories. To address these challenges, various dynamic control strategies have been developed to optimize the interplay between these two processes, such as quorum sensing systems (Cao et al., 2023, Xu, 2018). However, the existing dynamic control tools still face some problems, including insufficient orthogonality, narrow dynamic ranges, and delayed response times. These restrictions emphasize the urgent need to develop dynamic control tools with programmability, fast response time, orthogonality and wide applicability. Such advancements hold paramount importance for optimizing the production efficiency of microbial cell factories.

Genetic code expansion (GCE) technology is one of the prominent synthetic biology technologies for site-specific incorporate of non-canonical amino acids (ncAAs) utilizing aminoacyl tRNA synthetases and corresponding tRNA orthogonal pairs (Fig. 7E) (Lateef et al., 2022, Tang et al., 2022). This method enables precise regulation of the expression levels of key enzymes influencing cell growth through the exogenous addition of ncAAs, thereby further balancing carbon flow allocation between cell growth and target product synthesis. Currently, over 300 ncAAs have been incorporated via aminoacyl-tRNA synthetase/tRNA pairs, expanding the versatility of proteins and the providing a variety options to control protein translation (Chen et al., 2022). Research developed an efficient biological production strategy based on GCE that integrates cell growth with biosynthetic balance engineering (GCE-CGBBE). This strategy involves titrating gene expression associated with cell growth and metabolic flux by constructing ncAAs-dependent expression patterns. Ultimately, this approach resulted in a significant increase in the production of *N*-acetylneuraminic acid (Tian et al., 2020). The significance of this study lies in its proposal of a novel and effective strategy for balancing metabolic flux allocation between cellular growth and target product synthesis. Through rational design of ncAA-dependent expression systems, the intracellular metabolic network can be precisely modulated to achieve enhanced production efficiency. Furthermore, this methodology demonstrates extensive application potential and could be extended to the biosynthesis of diverse bioactive compounds in medicinal plants. Collectively, GCE technologyand its derivative strategies warrant further in-depth investigation and development for broader biotechnological applications.

### 3.6 Modular co-culture system

The prominent progress of heterologous production of chemicals by synthetic biology strategy was achieved based on a single host cell in monoculture fermentation. For instance, studies achieved the production of liquiritigenin at a titer of 867.67 mg/L by engineering a single *S. cerevisiae* (Deng, Li, Li, & Zhou, 2025). Nevertheless, the metabolic capabilities of an individual microbial strain are naturally constrained, which creates difficulties in producing natural products that demand intricate and extended metabolic pathways. Therefore, modular co-culture systems have been developed as a promising approach (Fig. 7F).

Modular co-culture is a strategy that enhances metabolic pathway efficiency by decomposing biosynthetic pathways into distinct functional modules and distributing these modules across different microorganisms. This strategy offers significant advantages, including the reduction of metabolic burden on the host, providing optimal enzymatic catalytic environment, and facilitating utilization of unique properties and substrate specificity of diverse microorganisms. For example, the production of amorphadiene was enhanced to 71.74 mg/L through the co-culture system of *Y. lipolytica* strains Po1f and Po1g, subcellular localization of amorphadiene synthase (*ADS*) gene (encoding amorphadiene synthase) into the endoplasmic reticulum, and the co-utilization of mixed carbon source (Marsafari, Az, Dou, & Xu, 2022). Complex metabolic pathways pose significant limitations on the biosynthesis of amorphadiene, whereas modular co-culture offers an effective platform to enhance its production. Furthermore, based on the endogenous Ehrlich pathway for tyrosol biosynthesis in *S. cerevisiae* and the superior catalytic efficiency of hydroxylase from *E. coli*, research designed a co-culture system involving *S. cerevisiae* and *E. coli* to achieve *de novo* production of hydroxytyrosol and the yield of hydroxytyrosol can reach 435.32 mg/L (Liu et al., 2024). Meanwhile, these studies have also revealed several inherent limitations in the system, including challenges in achieving long-term stability of dynamic systems, maintaining controllable population ratios among constituent members, and regulating the transport of metabolic intermediates. Nevertheless, it is undeniable that microbial co-culture strategies can effectively address challenges such as incompatibility between genetic elements and complex or incompatible metabolic pathways in host cells. This approach holds promise as a critical strategy for synthesizing complex bioactive compounds and advancing synthetic biology.

## 4 Summary and outlook

Bioactive compounds in medicinal plants, including flavonoids, terpenoids, and alkaloids, exhibit remarkable pharmacological activities and play a crucial role in maintaining human health. As the pursuit of health and longevity intensifies, the demand for these bioactive compounds is on the rise. Nowadays, the bioactive compounds can be produced *de novo* through synthetic biology technologies by constructing heterologous metabolic pathways within microbial cell factories. Compared to traditional plant extraction and chemical synthesis, this technology is notably greener and safer, offering a sustainable production mode. In addition, it remains unaffected by the external environment and facilitates large-scale sustainable cultivation, aligning with contemporary principles of green, low-carbon, and high-quality development.

Currently, the production of medicinal bioactive compounds utilizing synthetic biology technology has achieved significant advancements, such as berberine (16.9 mg/L), (+)-borneol (753 mg/L), and *β*-elemene (4.7 g/L) (Jiao et al., 2024, Ye et al., 2023; Zhang et al., 2025b). However, several challenges remain to be addressed.

The elucidation of biosynthetic pathways for bioactive compounds in medicinal plants is critical for microbial cell factory development. Nonetheless, many of these pathways remain unidentified, creating a substantial barrier to their microbial recombination and synthesis. For instance, the critical oxidase (Taxane 9*α* hydroxylase 1, T9*α*H1) in the paclitaxel biosynthetic pathway was only identified recently (Jiang et al., 2024). Sequencing technologies have advanced substantially in recent years. This progress has facilitated the exploration of a growing number of medicinal plant genomes, thereby driving the expansion of omics databases. Despite this, unlike in microorganisms, the genes responsible for biosynthetic pathways of active compounds in most medicinal plants are not grouped into clusters within the genome (Bernal-Gallardo & de Folter, 2024). This adds complexity to the study of synthetic pathways and the recognition of functional components. However, integrating multi-omics data (genomics, transcriptomics, proteomics) with machine learning-based gene clustering prediction tools (e.g., plantiSMASH) could accelerate discovery of these non-clustered pathways, necessitating continued innovation in this domain.

Selecting an optimal chassis host for bioactive compound biosynthesis is crucial. Factors such as the completeness of genomic information, the availability of genetic tools, and the safety of chassis strains, need to be carefully evaluated, especially in the contexts of pharmaceutical or food additive production. Currently, *E. coli*, *B. subtilis*, *C. glutamicum*, and *S. cerevisiae* are the most widely utilized chassis strains. They share common advantages including well-characterized genetic backgrounds, ease of genetic modification, and thoroughly elucidated metabolic networks, making them ideal platforms for constructing efficient heterologous expression systems. Notably, distinct application scenarios exist among these hosts. For instance, *E. coli* exhibits the fastest growth rate and highest fermentation density. However, the presence of endotoxins capable of inducing human immune responses necessitates complex separation processes for bioactive compound production, significantly increasing downstream purification costs (Ji et al., 2025). This requires careful consideration when employing *E. coli* in food and pharmaceutical applications. In contrast, *B. subtilis*, *C. glutamicum*, and *S. cerevisiae* have all obtained generally recognized as safe (GRAS) status, enabling their metabolic products to be directly applied in food and pharmaceutical sectors. Of greater significance, enzymes involved in metabolic pathways from medicinal plants, including cytochrome P450 and glycosyltransferase, tend to show limited expression in prokaryotic organisms such as *E. coli* and *B. subtilis* 168. However, these enzymes are effectively expressed in eukaryotic platforms, like yeast (Seshadri et al., 2025). Therefore, developing modular chassis screening platforms that evaluate host compatibility (e.g., P450 expression efficiency, GRAS compliance, metabolic burden) for target compound classes would streamline optimal strain selection.

The ongoing development of innovative synthetic biology regulatory tools are crucial for expediting the construction and optimization of microbial cell factories, which will enhance the biosynthesis of medicinal bioactive compounds. Conventional static regulatory approaches, such as gene knockouts and promoter replacements, predominantly focus on localized improvements. This can result in undesirable outcomes, including the excessive accumulation of intermediate metabolites, imbalances in metabolic flux, and cytotoxic effects. To mitigate these challenges, several dynamic control mechanisms have been developed. For example, a series of promoters with different induction strengths have been designed in *P. pastoris* to modulate the expression of target genes (Bai et al., 2025). Meanwhile, in *E. coli*, the construction of the salicylic acid-responsive biosensor system multiple antibiotic resistance regulator (MarR)-mar operon promoter (PmarO) biosensor system allowed dynamic regulation of the biosynthetic pathway of salicylic acid and its derivatives (Zou et al., 2021). Despite these advancements, these tools still encounter limitations, such as increased metabolic burdens on cells, delayed metabolic responses, and insufficient orthogonality. Therefore, there remains an urgent need to further develop more efficient and refined regulatory tools for synthetic biology applications.

The development of synthetic biology technology requires a large amount of funds and skilled talents. These high costs pose a heavy burden for many small and medium-sized companies and research institutions. At the same time, cultivating professional and technical personnel with cross-disciplinary knowledge (such as talents who master molecular biology, engineering and computer science simultaneously) is also an arduous task, and the scarcity of such high-end talents further aggravates the bottleneck of the industry. Cost efficiency represents another critical hurdle. While biomanufacturing enables the synthesis of certain compounds, it often proves more expensive than conventional plant extraction or chemical synthesis. For example, the rare molecule in cannabis, cannabidiol (CBD), has significant medical value. The cost of extracting CBD from cannabis is about $1 000−5 000 per kilogram (affected by planting density and extraction efficiency). In contrast, Ginkgo Bioworks has attempted to synthesize it using yeast, but the current cost exceeds $10 000 per kilogram, thus it has not been commercialized. This is mainly because the biological reaction process is complex, involving the regulation and optimization of multiple steps, and the efficiency of large-scale production has not yet reached the ideal level. Therefore, in the market competition, the price disadvantage of synthetic biology products makes it difficult to quickly occupy the mainstream market share. Another important issue is the strain robustness in industrial production. Engineering strains that perform well under laboratory conditions may experience performance degradation during large-scale fermentation due to factors such as environmental changes, metabolic stress, or gene mutations. This instability directly affects production efficiency and product quality, increasing the continuous investment of additional time and resources. For example, the American company Amyris attempted to produce artemisinin using yeast. Although the yield met the standard in the laboratory stage, the cost was out of control due to cell stress after scale-up, and eventually, industrialization was abandoned. To sum up, the development potential of synthetic biology is huge, but in practical applications, various obstacles still need to be overcome. Only through technological innovation, policy support, and industrial collaboration can the research and development costs be gradually reduced, production efficiency be improved, and ultimately, synthetic biology technology be promoted to mature and be widely applied in various fields. For example, to address these dual challenges of cost and technical complexity, establishing public–private partnerships for shared bioreactor facilities and developing automated AI-driven strain engineering platforms (e.g., cloud labs) could reduce financial barriers, mitigate talent shortages, and accelerate scalable bioprocess optimization.

In the future, synthetic biology technology will undoubtedly continue to play a crucial role and make even greater contributions to the development and advancement of human society. It is proposed that the following aspects could be prioritized for further research.

Constructing efficient microbial cell factories requires more than just optimizing biosynthetic pathways and reconfiguring cellular metabolism, it also calls for the creation of highly effective and precise genetic engineering tools. The CRISPR/Cas system has emerged as the leading gene-editing platform, allowing for accurate and diverse modifications throughout the genome (Guan et al., 2024). Despite its advantages, several challenges persist, such as limited editing efficiency, reliance on PAM sequences, potential off-target effects, and possible toxicity of Cas proteins (Lv et al., 2023). In this regard, protein engineering presents a viable solution to improve the cutting activity, specificity, and safety of Cas proteins, thereby enhancing the overall effectiveness of the CRISPR/Cas system. Furthermore, the progress in artificial intelligence has greatly enhanced the process of *de novo* protein design (Winnifrith et al., 2024). As a result, it is feasible to expand the application range of the editing system by modifying Cas proteins to have specific functions.

Create efficient, precise and flexible synthetic biology regulatory tools. The field of synthetic biology has made significant strides in recent years, but the development of advanced regulatory tools remains a critical area for improvement. Existing metabolic regulation methods predominantly focus on alterations at the transcriptional level, which often encounter several challenges. These include slow response times, limited orthogonality (the ability of a regulatory system to function independently without interfering with other cellular processes), and insufficient regulatory accuracy. As a result, there is an urgent need to create synthetic biology regulatory tools that can respond rapidly to changes in environmental or intracellular conditions. In addition to improving transcriptional-level regulation, strategies for protein-level regulation have demonstrated considerable promise, such as protein degradation tags and Genetic codon expansion technology (Chen et al., 2019; Tian et al., 2020). Protein degradation tags allow for the targeted removal of specific proteins within cells, providing high orthogonality and quick responsiveness. This ensures that only the desired proteins are degraded, minimizing unintended effects on cellular metabolism. Genetic codon expansion technology, on the other hand, enables the incorporation of non-canonical amino acids into proteins, offering new possibilities for controlling protein activity, stability, and localization. All in all, these techniques provide benefits like high orthogonality, quick responsiveness, and reduced metabolic load. For example, in yeast cells, combining phosphorylation-based regulation (for rapid response) with transcriptional regulation (for slower response) facilitated the development of a dual-time-scale dynamic regulatory system, enabling precise control over cellular activities (Gordley, Williams, Bashor, Toettcher, Yan, & Lim, 2016).

Synthetic biology focuses on the creation and design of biological tools and systems, which can involve in altering existing organisms by introducing novel characteristics or constructing entirely new organisms using non-living materials (Kurtoğlu, Yıldız, & Arda, 2024). Therefore, throughout this process, it is crucial to carefully examine and address ethical and safety issues. For example, research proposed a synthetic auxotrophy-based biocontainment strategy for yeast. By disrupting escape mechanisms through amber-suppressor tRNAs and incorporating a dual transcriptional-translational control switch, this approach constructs multiplex safeguard strains with robust growth and an escape frequency below 1 × 10^−9^. This system effectively prevents strain proliferation in real fermentation scenarios, offering a reliable solution for biosafety (Chang et al., 2023). Likewise, the synthetic yeast project not only highlighted successful international scientific cooperation but also emphasized the necessity of integrating ethical considerations with scientific progress, resulting in the establishment of guidelines aimed at minimizing biosafety risks (Zhao et al., 2023). The International Genetically Engineered Machine (iGEM) competition also requires participants to consider the broader societal implications of their projects from the outside. Additionally, the practical applications of synthetic biology must adhere to the regulatory frameworks and policies of different nations to ensure they are implemented legally and in accordance with established standards. Therefore, as synthetic biology continues to advance, the ethical and safety challenges it raises will remain a critical focus, demanding ongoing dialogue, adaptive governance, and proactive risk management strategies.

In conclusion, driven by continuous advancements in biotechnology and the emergence of novel synthetic biology regulatory tools, the efficient and high-yield biosynthesis of a broader spectrum of pharmaceutically active bioactive compounds in microbial cell factories is becoming increasingly feasible. This progress depends not only on core technological developments in genetic editing and metabolic engineering but also benefits from the maturation of multi-omics data analysis and high-throughput screening platforms. For instance, systematic optimization of microbial metabolic pathways has demonstrated significant improvements in target compound yields. Meanwhile, advanced fermentation process control strategies have proven effective in enhancing both production efficiency and product purity. Notably, this discussion of the ‘microbial cell factory’ concept specifically underscores the critical importance of technological convergence. Artificial intelligence-driven design methodologies are revitalizing synthetic biology through machine learning algorithms that predict metabolic network behaviors, design optimal genetic circuits, and screen potential enzymatic catalysts. Such approaches substantially reduce experimental cycles and associated costs. This interdisciplinary collaboration model bridges biology, computer science, and chemical engineering, collectively advancing microbial cell factories toward intelligent and precision-oriented development. In future developments, increased participation of researchers and technical experts engage in this field, it is anticipate the development of increasingly diversified and functionally robust microbial cell factories. These innovations will provide sustained momentum for green biomanufacturing of medicinal plant-derived bioactive compounds, ultimately establishing a sustainable alternative to traditional extraction methods.

## CRediT authorship contribution statement

**Yingjun Liu:** Conceptualization, Visualization, Writing – original draft, Writing – review & editing. **Anying Ji:** Conceptualization, Visualization, Writing – review & editing. **Haiyang Jia:** Writing – review & editing. **Huan Sun:** Project administration, Funding acquisition, Supervision, Writing – review & editing.

## Declaration of competing interest

The authors declare that they have no known competing financial interests or personal relationships that could have appeared to influence the work reported in this paper.

## References

[b0005] Adedeji A.A., Babalola O.O. (2020). Secondary metabolites as plant defensive strategy: A large role for small molecules in the near root region. Planta.

[b0010] Agrawal A., Yang Z.L., Blenner M. (2023). Engineering *Yarrowia lipolytica* for the biosynthesis of geraniol. Metabolic Engineering Communications.

[b0020] An T., Lin G.Y., Liu Y., Qin L., Xu Y.Q., Feng X.D. (2023). *De novo* biosynthesis of anticarcinogenic icariin in engineered yeast. Metabolic Engineering.

[b0025] Bai F., Cai P., Yao L., Shen Y.W., Li Y.X., Zhou Y.J. (2025). Inducible regulating homologous recombination enables precise genome editing in *Pichia pastoris* without perturbing cellular fitness. Trends in Biotechnology.

[b0030] Baker J.J., Shi J., Wang S.Y., Mujica E.M., Bianco S., Capponi S. (2025). ML-enhanced peroxisome capacity enables compartmentalization of multienzyme pathway. Nature Chemical Biology.

[b0035] Bergman M.E., Dudareva N. (2024). Plant specialized metabolism: Diversity of terpene synthases and their products. Current Opinion in Plant Biology.

[b0040] Bergman M.E., Davis B., Phillips M.A. (2019). Medically useful plant terpenoids: Biosynthesis, occurrence, and mechanism of action. Molecules.

[b0045] Bernal-Gallardo J.J., de Folter S. (2024). Plant genome information facilitates plant functional genomics. Planta.

[b0050] Bird J.E., Marles-Wright J., Giachino A. (2022). A user’s guide to golden gate cloning methods and standards. ACS Synthetic Biology.

[b0055] Bradley S.A., Lehka B.J., Hansson F.G., Adhikari K.B., Rago D., Rubaszka P. (2023). Biosynthesis of natural and halogenated plant monoterpene indole alkaloids in yeast. Nature Chemical Biology.

[b0060] Brázda V., Bartas M., Bowater R.P. (2021). Evolution of diverse strategies for promoter regulation. Trends in Genetics.

[b0065] Bui V.H., Rodríguez-López C.E., Dang T.T. (2023). Integration of discovery and engineering in plant alkaloid research: Recent developments in elucidation, reconstruction, and repurposing biosynthetic pathways. Current Opinion in Plant Biology.

[b0070] Buranasudja V., Rani D., Malla A., Kobtrakul K., Vimolmangkang S. (2021). Insights into antioxidant activities and anti-skin-aging potential of callus extract from *Centella asiatica* (L.). Scientific Reports.

[b0075] Cai G., Lin Z.Q., Shi S.B. (2022). Development and expansion of the CRISPR/Cas9 toolboxes for powerful genome engineering in yeast. Enzyme and Microbial Technology.

[b0080] Câmara J.S., Perestrelo R., Ferreira R., Berenguer C.V., Pereira J.A.M., Castilho P.C. (2024). Plant-derived terpenoids: A plethora of bioactive compounds with several health functions and industrial applications-a comprehensive overview. Molecules.

[b0085] Cao Z.N., Liu Z., Mao X.Z. (2023). Application of quorum sensing in metabolic engineering. Journal of Agricultural and Food Chemistry.

[b0090] Cazier A.P., Blazeck J. (2021). Advances in promoter engineering: Novel applications and predefined transcriptional control. Biotechnology Journal.

[b0095] Chai M., Deng C., Chen Q., Lu W., Liu Y.F., Li J.H. (2021). Synthetic biology toolkits and metabolic engineering applied in *Corynebacterium glutamicum* for biomanufacturing. ACS Synthetic Biology.

[b0105] Chang T.T., Ding W.C., Yan S.R., Wang Y., Zhang H.L., Zhang Y. (2023). A robust yeast biocontainment system with two-layered regulation switch dependent on unnatural amino acid. Nature Communications.

[b0130] Chen R.P., Gaynor A.S., Chen W. (2019). Synthetic biology approaches for targeted protein degradation. Biotechnology Advances.

[b0120] Chen H.N., Zhong L., Zhou H.B., Bai X.P., Sun T., Wang X.Y. (2023). Biosynthesis and engineering of the nonribosomal peptides with a C-terminal putrescine. Nature Communications.

[b0125] Chen K., Hu Z.M., Song W., Wang Z.L., He J.B., Shi X.M. (2019). Diversity of *O*-glycosyltransferases contributes to the biosynthesis of flavonoid and triterpenoid glycosides in *Glycyrrhiza uralensis*. ACS Synthetic Biology.

[b0135] Chen Y.D., Jin S.K., Zhang M.X., Hu Y., Wu K.L., Chung A. (2022). Unleashing the potential of noncanonical amino acid biosynthesis to create cells with precision tyrosine sulfation. Nature Communications.

[b0140] Cheng A.X., Lou Y.G., Mao Y.B., Lu S., Wang L.J., Chen X.Y. (2007). Plant terpenoids: Biosynthesis and ecological functions. Journal of Integrative Plant Biology.

[b0145] Costa C.E., Møller-Hansen I., Romaní A., Teixeira J.A., Borodina I., Domingues L. (2021). Resveratrol production from hydrothermally pretreated *Eucalyptus* wood using recombinant industrial *Saccharomyces cerevisiae* strains. ACS Synthetic Biology.

[b0150] Cravens A., Payne J., Smolke C.D. (2019). Synthetic biology strategies for microbial biosynthesis of plant natural products. Nature Communications.

[b0155] Cui S.X., Lv X.Q., Wu Y.K., Li J.H., Du G.C., Ledesma-Amaro R. (2019). Engineering a bifunctional Phr60-Rap60-Spo0A quorum-sensing molecular switch for dynamic fine-tuning of menaquinone-7 synthesis in *Bacillus subtilis*. ACS Synthetic Biology.

[b0160] Curran K.A., Karim A.S., Gupta A., Alper H.S. (2013). Use of expression-enhancing terminators in *Saccharomyces cerevisiae* to increase mRNA half-life and improve gene expression control for metabolic engineering applications. Metabolic Engineering.

[b0165] Curran K.A., Morse N.J., Markham K.A., Wagman A.M., Gupta A., Alper H.S. (2015). Short synthetic terminators for improved heterologous gene expression in yeast. ACS Synthetic Biology.

[b0170] De Nijs Y., De Maeseneire S.L., Soetaert W.K. (2020). 5’ untranslated regions: The next regulatory sequence in yeast synthetic biology. Biological Reviews of the Cambridge Philosophical Society.

[b0175] Debnath B., Singh W.S., Das M., Goswami S., Singh M.K., Maiti D. (2018). Role of plant alkaloids on human health: A review of biological activities. Materials Today Chemistry.

[b0185] Deng H.N., Li H.B., Li S., Zhou J.W. (2025). Engineering *Saccharomyces cerevisiae* for efficient liquiritigenin production. Journal of Agricultural and Food Chemistry.

[b0190] Di X.N., Ortega-Alarcon D., Kakumanu R., Iglesias-Fernandez J., Diaz L., Baidoo E.E.K. (2023). MEP pathway products allosterically promote monomerization of deoxy-*D*-xylulose-5-phosphate synthase to feedback-regulate their supply. Plant Communications.

[b0195] Dinh C.V., Prather K.L.J. (2019). Development of an autonomous and bifunctional quorum-sensing circuit for metabolic flux control in engineered *Escherichia coli*. Proceedings of the National Academy of Sciences of the United States of America.

[b0200] Duan L.J., Ding W.T., Liu X.N., Cheng X.Z., Cai J., Hua E.B. (2017). Biosynthesis and engineering of kaempferol in *Saccharomyces cerevisiae*. Microbial Cell Factories.

[b0210] Facchini P.J., De Luca V. (2008). Opium poppy and Madagascar periwinkle: Model non-model systems to investigate alkaloid biosynthesis in plants. Plant Journal.

[b0215] Faure G., Ogurtsov A.Y., Shabalina S.A., Koonin E.V. (2016). Role of mRNA structure in the control of protein folding. Nucleic Acids Research.

[b0220] Fordjour E., Liu C.L., Hao Y.P., Sackey I., Yang Y.K., Liu X.X. (2023). Engineering *Escherichia coli* BL21 (DE3) for high-yield production of germacrene A, a precursor of *β*-elemene via combinatorial metabolic engineering strategies. Biotechnology and Bioengineering.

[b0225] Galanie S., Thodey K., Trenchard I.J., Filsinger Interrante M., Smolke C.D. (2015). Complete biosynthesis of opioids in yeast. Science.

[b0230] Gao J.C., Zuo Y.M., Xiao F., Wang Y.L., Li D.F., Xu J.H. (2023). Biosynthesis of catharanthine in engineered *Pichia pastoris*. Nature Synthesis.

[b0235] Gao S.L., Tong Y.Y., Zhu L., Ge M., Zhang Y.A., Chen D.J. (2017). Iterative integration of multiple-copy pathway genes in *Yarrowia lipolytica* for heterologous *β*-carotene production. Metabolic Engineering.

[b0240] García-Nafría J., Watson J.F., Greger I.H. (2016). IVA cloning: A single-tube universal cloning system exploiting bacterial *in vivo* assembly. Scientific Reports.

[b0245] Gayen A.K., Nichols L., Williams G.J. (2020). An artificial pathway for polyketide biosynthesis. Nature Catalysis.

[b0255] Gibson D.G., Young L., Chuang R.Y., Venter J.C., Hutchison C.A., Smith H.O. (2009). Enzymatic assembly of DNA molecules up to several hundred kilobases. Nature Methods.

[b0265] Glaser J.A. (2016). Synthetic biology leading to specialty chemicals. Clean Technologies and Environmental Policy.

[b0270] Glasscock C.J., Biggs B.W., Lazar J.T., Arnold J.H., Burdette L.A., Valdes A. (2021). Dynamic control of gene expression with riboregulated switchable feedback promoters. ACS Synthetic Biology.

[b0275] Gordley R.M., Williams R.E., Bashor C.J., Toettcher J.E., Yan S.D., Lim W.A. (2016). Engineering dynamical control of cell fate switching using synthetic phospho-regulons. Proceedings of the National Academy of Sciences of the United States of America.

[b0280] Gou Y.W., Li D.F., Zhao M.H., Li M.X., Zhang J.J., Zhou Y.L. (2024). Intein-mediated temperature control for complete biosynthesis of sanguinarine and its halogenated derivatives in yeast. Nature Communications.

[b0285] Guan A.L., He Z.X., Wang X., Jia Z.J., Qin J.F. (2024). Engineering the next-generation synthetic cell factory driven by protein engineering. Biotechnology Advances.

[b0290] Guan Z.H., Yao N.Y., Yuan W.L., Li F.L., Xiao Y., Rehmutulla M. (2025). Total biosynthesis of cotylenin diterpene glycosides as 14-3-3 protein-protein interaction stabilizers. Chemical Science.

[b0295] Guo G.Y., Yu H.Z., Wang H., Wang P., Liu J.X., Battseren T. (2024). Visualized analysis of licorice research hotspots and trends in the field of traditional Chinese medicine resources based on VOSviewer and CiteSpace knowledge maps. Food & Medicine Homology.

[b0300] Han Y.H., Kim G., Seo S.W. (2023). Programmable synthetic biology tools for developing microbial cell factories. Current Opinion in Biotechnology.

[b0305] He B., Ma Y., Tian F.F., Zhao G.R., Wu Y., Yuan Y.J. (2023). YLC-assembly: Large DNA assembly via yeast life cycle. Nucleic Acids Research.

[b0310] He Q., Yue M.Y., Liu M.S., Ren X.F., Wang H.J., Chen J.B. (2025). *De novo* biosynthesis of dihydroquercetin in an engineered *Yarrowia lipolytica* with enhanced (2S)-eriodictyol. Food Bioscience.

[b0315] Hong X.K., Guo T.L., Xu X.Q., Lin J. (2023). Multiplex metabolic pathway engineering of *Monascus pilosus* enhances lovastatin production. Applied Microbiology and Biotechnology.

[b0325] Huang Y.L., Ye Z.L., Wan X.K., Yao G., Duan J.Y., Liu J.J. (2023). Systematic mining and evaluation of the sesquiterpene skeletons as high energy aviation fuel molecules. Advanced Science.

[b0335] Hwang H.G., Milito A., Yang J.S., Jang S., Jung G.Y. (2023). Riboswitch-guided *Chalcone* synthase engineering and metabolic flux optimization for enhanced production of flavonoids. Metabolic Engineering.

[b0340] Ito Y., Terai G., Ishigami M., Hashiba N., Nakamura Y., Bamba T. (2020). Exchange of endogenous and heterogeneous yeast terminators in *Pichia pastoris* to tune mRNA stability and gene expression. Nucleic Acids Research.

[b0350] Jamieson C.S., Misa J., Tang Y., Billingsley J.M. (2021). Biosynthesis and synthetic biology of psychoactive natural products. Chemical Society Reviews.

[b0355] Jensen D., Galburt E.A. (2021). The context-dependent influence of promoter sequence motifs on transcription initiation kinetics and regulation. Journal of Bacteriology.

[b0360] Jha Y., Mohamed H.I. (2022). Plant secondary metabolites as a tool to investigate biotic stress tolerance in plants: A review. Gesunde Pflanzen.

[b0365] Ji A.Y., Zou D., Ma A.M., Wei X.T. (2025). Rational design of DAHP synthase and prephenate dehydrogenase for metabolic engineering of *Bacillus amyloliquefaciens* to produce *L*-tyrosine. International Journal of Biological Macromolecules.

[b0370] Ji D.N., Li J.H., Xu F.L., Ren Y.H., Wang Y. (2021). Improve the biosynthesis of baicalein and scutellarein via manufacturing self-assembly enzyme reactor *in vivo*. ACS Synthetic Biology.

[b0380] Jiang D.H., Yang M.Q., Chen K., Jiang W.X., Zhang L.L., Ji X.J. (2024). Exploiting synthetic biology platforms for enhanced biosynthesis of natural products in *Yarrowia lipolytica*. Bioresource Technology.

[b0385] Jiang G.Z., Yao M.D., Wang Y., Zhou L., Song T.Q., Liu H. (2017). Manipulation of GES and ERG20 for geraniol overproduction in *Saccharomyces cerevisiae*. Metabolic Engineering.

[b0390] Jiang T., Li C.Y., Zou Y.S., Zhang J.L., Gan Q., Yan Y.J. (2022). Establishing an Autonomous Cascaded Artificial Dynamic (AutoCAD) regulation system for improved pathway performance. Metabolic Engineering.

[b0395] Jiao X., Fu X.Z., Li Q.S., Bu J.L., Liu X.Y., Savolainen O. (2024). *De novo* production of protoberberine and benzophenanthridine alkaloids through metabolic engineering of yeast. Nature Communications.

[b0405] Kim E.M., Woo H.M., Tian T., Yilmaz S., Javidpour P., Keasling J.D. (2017). Autonomous control of metabolic state by a quorum sensing (QS)-mediated regulator for bisabolene production in engineered *E*. *coli*. Metabolic Engineering.

[b0415] Kong X., Wu Y.K., Yu W.W., Liu Y.F., Li J.H., Du G.C. (2023). Efficient synthesis of limonene in *Saccharomyces cerevisiae* using combinatorial metabolic engineering strategies. Journal of Agricultural and Food Chemistry.

[b0420] Kowalczyk S., Grymel M., Bilik J., Kula W., Wawoczny A., Grymel P. (2024). Selected plants as sources of natural and active ingredients for cosmetics of the future. Applied Sciences.

[b0425] Kurtoğlu A., Yıldız A., Arda B. (2024). The view of synthetic biology in the field of ethics: A thematic systematic review. Frontiers in Bioengineering and Biotechnology.

[b0435] Kuzuyama, T., & Seto, H. (2012). Two distinct pathways for essential metabolic precursors for isoprenoid biosynthesis. *Proceedings of the Japan Academy Series B, Physical and Biological Sciences*, *88*(3), 41–52. 10.2183/pjab.88.41 PMC3365244 22450534

[b0440] Lateef O.M., Akintubosun M.O., Olaoba O.T., Samson S.O., Adamczyk M. (2022). Making sense of “nonsense” and more: Challenges and opportunities in the genetic code expansion, in the world of tRNA modifications. International Journal of Molecular Sciences.

[b0445] Lee S.H., Hu Y., Chou A., Chen J., Gonzalez R. (2024). Metabolic flux optimization of iterative pathways through orthogonal gene expression control: Application to the *β*-oxidation reversal. Metabolic Engineering.

[b0450] Lei D.W., Qiu Z.T., Wu J.H., Qiao B., Qiao J.J., Zhao G.R. (2021). Combining metabolic and monoterpene synthase engineering for *de novo* production of monoterpene alcohols in *Escherichia coli*. ACS Synthetic Biology.

[b0460] Li J.J., Gao J.Q., Ye M., Cai P., Yu W., Zhai X.X. (2024). Engineering yeast for high-level production of *β*-farnesene from sole methanol. Metabolic Engineering.

[b0465] Li J.Z., Wang X.X., Xokat X., Wan Y., Gao X.P., Wang Y. (2025). Metabolic engineering of *Corynebacterium glutamicum* for producing different types of triterpenoids. ACS Synthetic Biology.

[b0475] Li L., Deng A.H., Liu S.W., Wang J.Y., Shi R.L., Wang T.T. (2022). A universal method for developing autoinduction expression systems using AHL-mediated quorum-sensing circuits. ACS Synthetic Biology.

[b0480] Li M.Z., Elledge S.J. (2007). Harnessing homologous recombination *in vitro* to generate recombinant DNA via SLIC. Nature Methods.

[b0485] Li Z.G., Wang Y.X., Xu M.W., Liu H.Y., Li L., Xu D.L. (2023). Molecular mechanism overview of metabolite biosynthesis in medicinal plants. Plant Physiology and Biochemistry.

[b0490] Liang J., Liu Z.H., Low X.Z., Ang E.L., Zhao H.M. (2017). Twin-primer non-enzymatic DNA assembly: An efficient and accurate multi-part DNA assembly method. Nucleic Acids Research.

[b0500] Lim S.H., Baek J.I., Jeon B.M., Seo J.W., Kim M.S., Byun J.Y. (2021). CRISPRi-guided metabolic flux engineering for enhanced protopanaxadiol production in *Saccharomyces cerevisiae*. International Journal of Molecular Sciences.

[b0505] Lim S.R., Lee S.J. (2024). Multiplex CRISPR-cas genome editing: Next-generation microbial strain engineering. Journal of Agricultural and Food Chemistry.

[b0515] Liu Q.L., Liu Y., Li G., Savolainen O., Chen Y., Nielsen J. (2021). *De novo* biosynthesis of bioactive isoflavonoids by engineered yeast cell factories. Nature Communications.

[b0520] Liu T.F., Gou Y.W., Zhang B., Gao R., Dong C., Qi M.M. (2022). Construction of ajmalicine and sanguinarine *de novo* biosynthetic pathways using stable integration sites in yeast. Biotechnology and Bioengineering.

[b0535] Liu X.N., Ding W.T., Jiang H.F. (2017). Engineering microbial cell factories for the production of plant natural products: From design principles to industrial-scale production. Microbial Cell Factories.

[b0560] Liu Y.Z., Zhao X.X., Gan F., Chen X.Y., Deng K., Crowe S.A. (2024). Complete biosynthesis of QS-21 in engineered yeast. Nature.

[b0565] Lv X.Q., Li Y., Xiu X., Liao C., Xu Y.M., Liu Y.F. (2023). CRISPR genetic toolkits of classical food microorganisms: Current state and future prospects. Biotechnology Advances.

[b0570] Lv Y.K., Edwards H., Zhou J.W., Xu P. (2019). Combining 26s rDNA and the cre-loxP system for iterative gene integration and efficient marker curation in *Yarrowia lipolytica*. ACS Synthetic Biology.

[b0575] Lv Y.K., Marsafari M., Koffas M., Zhou J.W., Xu P. (2019). Optimizing oleaginous yeast cell factories for flavonoids and hydroxylated flavonoids biosynthesis. ACS Synthetic Biology.

[b0585] Mao J.W., Mohedano M.T., Fu J., Li X.W., Liu Q.L., Nielsen J. (2023). Fine-tuning of *p*-coumaric acid synthesis to increase (2*S*)-naringenin production in yeast. Metabolic Engineering.

[b0590] Marchev A.S., Dinkova-Kostova A.T., György Z., Mirmazloum I., Aneva I.Y., Georgiev M.I. (2016). *Rhodiola rosea* L. From golden root to green cell factories. Phytochemistry Reviews.

[b0595] Marsafari M., Xu P. (2020). Debottlenecking mevalonate pathway for antimalarial drug precursor amorphadiene biosynthesis in *Yarrowia lipolytica*. Metabolic Engineering Communications.

[b0600] Marsafari M., Azi F., Dou S.H., Xu P. (2022). Modular co-culture engineering of *Yarrowia lipolytica* for amorphadiene biosynthesis. Microbial Cell Factories.

[b0605] Marsan C.B., Lee S.G., Nguyen A., Gordillo Sierra A.R., Coleman S.M., Brooks S.M. (2024). Leveraging a *Y. lipolytica* naringenin chassis for biosynthesis of apigenin and associated glucoside. Metabolic Engineering.

[b0610] Meinke G., Bohm A., Hauber J., Pisabarro M.T., Buchholz F. (2016). Cre recombinase and other tyrosine recombinases. Chemical Reviews.

[b0615] Mir A., Edraki A., Lee J., Sontheimer E.J. (2018). Type II-C CRISPR-Cas9 biology, mechanism, and application. ACS Chemical Biology.

[b0620] Mishra S.K., Tripathi G., Kishore N., Singh R.K., Singh A., Tiwari V.K. (2017). Drug development against tuberculosis: Impact of alkaloids. European Journal of Medicinal Chemistry.

[b0625] Moon J.H., Lee K., Lee J.H., Lee P.C. (2020). Redesign and reconstruction of a steviol-biosynthetic pathway for enhanced production of steviol in *Escherichia coli*. Microbial Cell Factories.

[b0630] Mózsik L., Hoekzema M., de Kok N.A.W., Bovenberg R.A.L., Nygård Y., Driessen A.J.M. (2021). CRISPR-based transcriptional activation tool for silent genes in filamentous fungi. Scientific Reports.

[b0635] Mutalik V.K., Guimaraes J.C., Cambray G., Lam C., Christoffersen M.J., Mai Q.A. (2013). Precise and reliable gene expression *via* standard transcription and translation initiation elements. Nature Methods.

[b0640] Ng Y.P., Or T.C.T., Ip N.Y. (2015). Plant alkaloids as drug leads for Alzheimer’s disease. Neurochemistry International.

[b0645] Nielsen J., Keasling J.D. (2016). Engineering cellular metabolism. Cell.

[b0650] Niu T.F., Huang C.K., Wang R.F., Yang L., Zhao S.J., Wang Z.T. (2024). Combinatorial metabolic engineering of *Bacillus subtilis* enables the efficient biosynthesis of isoquercitrin from quercetin. Microbial Cell Factories.

[b0655] Nomura Y., Seki H., Suzuki T., Ohyama K., Mizutani M., Kaku T. (2019). Functional specialization of UDP-glycosyltransferase 73P12 in licorice to produce a sweet triterpenoid saponin, glycyrrhizin. Plant Journal.

[b0660] Nose M., Yamanaka K., Hisaka S., Inui T., Kawano N., Hayashi S. (2019). Evaluation of the safety and efficacy of *Glycyrrhiza uralensis* root extracts produced using artificial hydroponic-field hybrid cultivation systems II: Comparison of serum concentration of glycyrrhetinic acid serum concentration in mice. Journal of Natural Medicines.

[b0670] Olorunniji F.J., Rosser S.J., Stark W.M. (2016). Site-specific recombinases: Molecular machines for the genetic revolution. Biochemical Journal.

[b0675] Pacesa M., Lin C.H., Cléry A., Saha A., Arantes P.R., Bargsten K. (2022). Structural basis for Cas9 off-target activity. Cell.

[b0680] Pacesa M., Pelea O., Jinek M. (2024). Past, present, and future of CRISPR genome editing technologies. Cell.

[b0685] Paddon C.J., Westfall P.J., Pitera D.J., Benjamin K., Fisher K., McPhee D. (2013). High-level semi-synthetic production of the potent antimalarial artemisinin. Nature.

[b0690] Panchal K., Tiwari A.K. (2017). *Drosophila melanogaster* “a potential model organism” for identification of pharmacological properties of plants/plant-derived components. Biomedicine & Pharmacotherapy.

[b0700] Parkan K., Pohl R., Kotora M. (2014). Cross-coupling reaction of saccharide-based alkenyl boronic acids with aryl halides: The synthesis of bergenin. Chemistry.

[b0705] Peng F., Wang X.Y., Sun Y., Dong G.B., Yang Y.K., Liu X.X. (2017). Efficient gene editing in *Corynebacterium glutamicum* using the CRISPR/Cas9 system. Microbial Cell Factories.

[b0710] Pomerantsev A.P., McCall R.M., Chahoud M., Hepler N.K., Fattah R., Leppla S.H. (2017). Genome engineering in *Bacillus anthracis* using tyrosine site-specific recombinases. PLoS One.

[b0715] Qian J.Y., Wang Y.Z., Hu Z.J., Shi T.Q., Wang Y.T., Ye C. (2023). *Bacillus* sp. as a microbial cell factory: Advancements and future prospects. Biotechnology Advances.

[b0720] Qiu N., Abegg D., Guidi M., Gilmore K., Seeberger P.H., Adibekian A. (2022). Artemisinin inhibits NRas palmitoylation by targeting the protein acyltransferase ZDHHC6. Cell Chemical Biology.

[b0730] Rana S., Upadhyay L.S.B. (2020). Microbial exopolysaccharides: Synthesis pathways, types and their commercial applications. International Journal of Biological Macromolecules.

[b0735] Rao X.L., Li D., Su Z.W., Nomura C.T., Chen S.W., Wang Q. (2024). A smart *RBS* library and its prediction model for robust and accurate fine-tuning of gene expression in *Bacillus* species. Metabolic Engineering.

[b0740] Rao Y., Yang J.Y., Wang J.Q., Yang X.Y., Zhang M.X., Zhan Y.Y. (2022). Minimization and optimization of *α*-amylase *Terminator* for heterologous protein production in *Bacillus licheniformis*. Bioresources and Bioprocessing.

[b0745] Ray-Soni A., Bellecourt M.J., Landick R. (2016). Mechanisms of bacterial transcription termination: All good things must end. Annual Review of Biochemistry.

[b0750] Ru, Z. Y., University, J., Liu, M. S., University, J., Chen, Q. H., University, J., et al. (2025). High-level *de novo* production of (2*S*)-naringenin in *Yarrowia lipolytica* using metabolic and enzyme engineering. *ACS Agricultural Science & Technology*, *5*(5), 784–793.

[b0755] Sachla A.J., Alfonso A.J., Helmann J.D. (2021). A simplified method for CRISPR-Cas9 engineering of *Bacillus subtilis*. Microbiology Spectrum.

[b0760] Sáez-Sáez J., Wang G.K., Marella E.R., Sudarsan S., Cernuda Pastor M., Borodina I. (2020). Engineering the oleaginous yeast *Yarrowia lipolytica* for high-level resveratrol production. Metabolic Engineering.

[b0765] Schläger S., Dräger B. (2016). Exploiting plant alkaloids. Current Opinion in Biotechnology.

[b0770] Seshadri K., Abad A.N.D., Nagasawa K.K., Yost K.M., Johnson C.W., Dror M.J. (2025). Synthetic biology in natural product biosynthesis. Chemical Reviews.

[b0775] Shamsudin N.F., Ahmed Q.U., Mahmood S., Ali Shah S.A., Khatib A., Mukhtar S. (2022). Antibacterial effects of flavonoids and their structure-activity relationship study: A comparative interpretation. Molecules.

[b0780] Shan M.Y., Yao M.D., Liang N., Wang H.R., Wu N., Wang Y. (2023). One-pot efficient bioconversion of crocetin from Zeaxanthin via a dual-enzyme system. ACS Sustainable Chemistry & Engineering.

[b0785] Shao Z.Y., Zhao H., Zhao H.M. (2009). DNA assembler, an *in vivo* genetic method for rapid construction of biochemical pathways. Nucleic Acids Research.

[b0790] Shen N., Wang T.F., Gan Q., Liu S.A., Wang L., Jin B. (2022). Plant flavonoids: Classification, distribution, biosynthesis, and antioxidant activity. Food Chemistry.

[b0800] Sheng N., Zhang Z.H., Zheng H., Ma C.Y., Li M.L., Wang Z. (2023). Scutellarin rescued mitochondrial damage through ameliorating mitochondrial glucose oxidation via the pdk-pdc axis. Advanced Science.

[b0810] Siedler S., Khatri N.K., Zsohár A., Kjærbølling I., Vogt; M., Hammar P. (2017). Development of a bacterial biosensor for rapid screening of yeast *p*-coumaric acid production. ACS Synthetic Biology.

[b0815] Sreekanth V., Zhou Q.X., Kokkonda P., Bermudez-Cabrera H.C., Lim D., Law B.K. (2020). Chemogenetic system demonstrates that Cas9 longevity impacts genome editing outcomes. ACS Central Science.

[b0820] Srinivasan P., Smolke C.D. (2020). Biosynthesis of medicinal tropane alkaloids in yeast. Nature.

[b0825] Sun W.T., Wan S.T., Liu C.Y., Wang R.W., Zhang H.C., Qin L. (2024). Establishing cell suitability for high-level production of licorice triterpenoids in yeast. Acta Pharmaceutica Sinica B.

[b0830] Sun W.Z., Wang X., Fu M.Y., Liu L.F., Zhang P., Yin B.C. (2025). Metabolic engineering of *Yarrowia lipolytica* for enhanced *de novo* biosynthesis of icaritin. ACS Synthetic Biology.

[b0840] Taha H.S. (2003). Effect of biotic stress (*Aspergillus niger*) on the production and accumulation of total alkaloids in *Atropa belladonna* L. via tissue culture. Acta Horticulturae.

[b0845] Tan X.J., Xiao Z.Q., Zhang S.Q., Wang Y.T., Zhao Y.F., Lu Q.Y. (2025). Engineering *Saccharomyces cerevisiae* for *De novo* biosynthesis of 3’-hydroxygenistein. Journal of Agricultural and Food Chemistry.

[b0850] Tang H.T., Zhang P., Luo X.Z. (2022). Recent technologies for genetic code expansion and their implications on synthetic biology applications. Journal of Molecular Biology.

[b0855] Taroncher M., Vila-Donat P., Tolosa J., Ruiz M.J., Rodríguez-Carrasco Y. (2021). Biological activity and toxicity of plant nutraceuticals: An overview. Current Opinion in Food Science.

[b0860] Tartik M., Liu J., Mohedano M.T., Mao J.W., Chen Y. (2023). Optimizing yeast for high-level production of kaempferol and quercetin. Microbial Cell Factories.

[b0865] Taylor G.M., Mordaka P.M., Heap J.T. (2019). Start-Stop Assembly: A functionally scarless DNA assembly system optimized for metabolic engineering. Nucleic Acids Research.

[b0870] TerMaat J.R., Pienaar E., Whitney S.E., Mamedov T.G., Subramanian A. (2009). Gene synthesis by integrated polymerase chain assembly and PCR amplification using a high-speed thermocycler. Journal of Microbiological Methods.

[b0875] Tian R.Z., Liu Y.F., Cao Y.T., Zhang Z.J., Li J.H., Liu L. (2020). Titrating bacterial growth and chemical biosynthesis for efficient *N*-acetylglucosamine and *N*-acetylneuraminic acid bioproduction. Nature Communications.

[b0885] Tian W.Y., Sun C.H., Zheng M., Harmer J.R., Yu M.J., Zhang Y.N. (2018). Efficient biosynthesis of heterodimeric C3-aryl pyrroloindoline alkaloids. Nature Communications.

[b0890] Tong Y.J., Li N., Zhou S.H., Zhang L., Xu S., Zhou J.W. (2024). Improvement of *Chalcone* synthase activity and high-efficiency fermentative production of (2*S*)-naringenin via *in vivo* biosensor-guided directed evolution. ACS Synthetic Biology.

[b0895] Tong Y.J., Zhou J.W., Zhang L., Xu P. (2021). A golden-gate based cloning toolkit to build violacein pathway libraries in *Yarrowia lipolytica*. ACS Synthetic Biology.

[b0900] Vadhel A., Bashir S., Mir A.H., Girdhar M., Kumar D., Kumar A. (2023). Opium alkaloids, biosynthesis, pharmacology and association with cancer occurrence. Open Biology.

[b0910] Volke D.C., Rohwer J., Fischer R., Jennewein S. (2019). Investigation of the methylerythritol 4-phosphate pathway for microbial terpenoid production through metabolic control analysis. Microbial Cell Factories.

[b0920] Wang P.P., Li C.J., Li X.D., Huang W.J., Wang Y., Wang J.L. (2021). Complete biosynthesis of the potential medicine icaritin by engineered *Saccharomyces cerevisiae* and *Escherichia coli*. Science Bulletin.

[b0925] Wang S.C., Alseekh S., Fernie A.R., Luo J. (2019). The structure and function of major plant metabolite modifications. Molecular Plant.

[b0930] Wang S., Lin S.X., Fang Q., Gyampoh R., Lu Z., Gao Y.L. (2022). A ribosomally synthesised and post-translationally modified peptide containing a *β*-enamino acid and a macrocyclic motif. Nature Communications.

[b0935] Wang Y.F., Wang G., Liu Y.P., Yang F.Y., Zhang H.S., Kong Y. (2023). Icaritin inhibits endometrial carcinoma cells by suppressing O-GlcNAcylation of FOXC1. Phytomedicine.

[b0940] Wang Y.T., Xiao Z.Q., Zhang S.Q., Tan X.J., Zhao Y.F., Liu J. (2024). Systematic engineering of *Saccharomyces cerevisiae* for the *de novo* biosynthesis of genistein and glycosylation derivatives. Journal of Fungi.

[b0945] Wei C.L., Zhang L.H., Shen W., Zou W., Xia Y.Y., Chen X.Z. (2024). Enhancement of squalene synthesis in *Candida tropicalis* via combinatorial metabolic engineering strategies to rebuild pathways. Biochemical Engineering Journal.

[b0950] Wei W.P., Zhang P., Shang Y.Z., Zhou Y., Ye B.C. (2020). Metabolically engineering of *Yarrowia lipolytica* for the biosynthesis of naringenin from a mixture of glucose and xylose. Bioresource Technology.

[b0955] Wei W., Zeng Q.X., Wang Y., Guo X.X., Fan T.Y., Li Y.H. (2023). Discovery and identification of EIF2AK2 as a direct key target of berberine for anti-inflammatory effects. Acta Pharmaceutica Sinica B.

[b0960] Westbrook A.W., Moo-Young M., Chou C.P. (2016). Development of a CRISPR-Cas9 tool kit for comprehensive engineering of *Bacillus subtilis*. Applied and Environmental Microbiology.

[b0965] Winnifrith A., Outeiral C., Hie B.L. (2024). Generative artificial intelligence for *de novo* protein design. Current Opinion in Structural Biology.

[b0970] Wu N., Pan Y., Liu Q., Shahidi F., Li H.Y., Chen F. (2025). Protective benefits and mechanisms of *Phyllanthus emblica* Linn. on aging induced by oxidative stress: A system review. Food & Medicine Homology.

[b0975] Wu X., Liu J.Y., Liu D., Yuwen M.M., Koffas M.A.G., Zha J. (2022). Biosynthesis of eriodictyol from tyrosine by *Corynebacterium glutamicum*. Microbial Cell Factories.

[b0980] Xia J.X., Ma S.J., Zhu X., Chen C., Zhang R., Cao Z.L. (2022). Versatile ginsenoside Rg3 liposomes inhibit tumor metastasis by capturing circulating tumor cells and destroying metastatic niches. Science Advances.

[b0985] Xiao F.X., Zhang Y.P., Zhang L.H., Li S.Y., Chen W., Shi G.Y. (2024). Advancing *Bacillus licheniformis* as a superior expression platform through promoter engineering. Microorganisms.

[b0990] Xiao Z.Q., Wang Y.T., Liu J., Zhang S.Q., Tan X.J., Zhao Y.F. (2023). Systematic engineering of *Saccharomyces cerevisiae* chassis for efficient flavonoid-7-*O*-disaccharide biosynthesis. ACS Synthetic Biology.

[b0995] Xu K., Zhao Y.J., Ahmad N., Wang J.N., Lv B., Wang Y. (2021). *O*-Glycosyltransferases from *Homo* sapiens contributes to the biosynthesis of glycyrrhetic acid 3-*O*-mono-*β*-*D*-glucuronide and glycyrrhizin in *Saccharomyces cerevisiae*. Synthetic and Systems Biotechnology.

[b1000] Xu M., Yang N., Pan J., Hua Q., Li C.X., Xu J.H. (2024). Remodeling the homologous recombination mechanism of *Yarrowia lipolytica* for high-level biosynthesis of squalene. Journal of Agricultural and Food Chemistry.

[b1005] Xu P. (2018). Production of chemicals using dynamic control of metabolic fluxes. Current Opinion in Biotechnology.

[b1015] Yamanishi M., Ito Y., Kintaka R., Imamura C., Katahira S., Ikeuchi A. (2013). A genome-wide activity assessment of *Terminator* regions in *Saccharomyces cerevisiae* provides a “Terminatome” toolbox. ACS Synthetic Biology.

[b1020] Yamazaki Y., Urano A., Sudo H., Kitajima M., Takayama H., Yamazaki M. (2003). Metabolite profiling of alkaloids and strictosidine synthase activity in camptothecin producing plants. Phytochemistry.

[b1030] Yan X., Yu H.J., Hong Q., Li S.P. (2008). Cre/lox system and PCR-based genome engineering in *Bacillus subtilis*. Applied and Environmental Microbiology.

[b1035] Yang B., Yang J.L., Zhao Y.P., Liu H.L., Jiang Y.M. (2016). The plant resources, structure characteristics, biological activities and synthesis of pyranoflavonoids. Current Medicinal Chemistry.

[b1040] Yang H., Song C.N., Liu C.W., Wang P.C. (2024). Synthetic biology tools for engineering *Aspergillus oryzae*. Journal of Fungi.

[b1050] Yang W.Q., Chen X., Li Y.L., Guo S.F., Wang Z., Yu X.L. (2020). Advances in pharmacological activities of terpenoids. Natural Product Communications.

[b1060] Yao H., Vu M.D., Liu X.W. (2019). Recent advances in reagent-controlled stereoselective/stereospecific glycosylation. Carbohydrate Research.

[b1070] Ye M., Gao J.Q., Zhou Y.J. (2023). Global metabolic rewiring of the nonconventional yeast *Ogataea polymorpha* for biosynthesis of the sesquiterpenoid *β*-elemene. Metabolic Engineering.

[b1075] Ye M., Gao J.Q., Li J.J., Yu W., Bai F., Zhou Y.J. (2024). Promoter engineering enables precise metabolic regulation towards efficient *β*-elemene production in *Ogataea polymorpha*. Synthetic and Systems Biotechnology.

[b1080] Yu B.J., Sung B.H., Koob M.D., Lee C.H., Lee J.H., Lee W.S. (2002). Minimization of the *Escherichia coli* genome using a Tn5-targeted Cre/loxP excision system. Nature Biotechnology.

[b1085] Yue M.Y., Liu M.S., Gao S., Ren X.F., Zhou S.H., Rao Y.J. (2024). High-level *de novo* production of (2*S*)-eriodictyol in *Yarrowia lipolytica* by metabolic pathway and NADPH regeneration engineering. Journal of Agricultural and Food Chemistry.

[b1090] Zhang C.T., Liu M.S., Wang X.L., Cheng J.Y., Xiang J.B., Yue M.Y. (2025). *De novo* synthesis of reticuline and taxifolin using re-engineered homologous recombination in *Yarrowia lipolytica*. ACS Synthetic Biology.

[b1095] Zhang H.Y., Cai P., Guo J., Gao J.Q., Xie L.F., Su P. (2025). Engineering cellular dephosphorylation boosts (+)-borneol production in yeast. Acta Pharmaceutica Sinica B.

[b1100] Zhang J., Hansen L.G., Gudich O., Viehrig K., Lassen L.M.M., Schrübbers L. (2022). A microbial supply chain for production of the anti-cancer drug vinblastine. Nature.

[b1110] Zhang Q., Wu Y.H., Huang X.S., Liu H.L., Wang Y. (2024). Design and optimization for efficient production of (*S*)-canadine in *Escherichia coli*. ACS Sustainable Chemistry & Engineering.

[b1115] Zhang Q., Yu S.Q., Lyu Y.B., Zeng W.Z., Zhou J.W. (2021). Systematically engineered fatty acid catabolite pathway for the production of (2*S*)-naringenin in *Saccharomyces cerevisiae*. ACS Synthetic Biology.

[b1140] Zhang Y.M., Wang G. (2025). A comparative study of goji berry and ashwagandha extracts effects on promoting neural stem cell proliferation and reducing neuronal cell death. Food & Medicine Homology.

[b1145] Zhang Y.P., Wang J., Wang Z.B., Zhang Y.M., Shi S.B., Nielsen J. (2019). A gRNA-tRNA array for CRISPR-Cas9 based rapid multiplexed genome editing in *Saccharomyces cerevisiae*. Nature Communications.

[b1155] Zhang Z., Lu Y., Chi Z., Liu G.L., Jiang H., Hu Z. (2019). Genome editing of different strains of *Aureobasidium melanogenum* using an efficient Cre/loxp site-specific recombination system. Fungal Biology.

[b1150] Zhang Z.H., Wu Q.Y., Ge Y., Huang Z.Y., Hong R., Li A.T. (2023). Hydroxylases involved in terpenoid biosynthesis: A review. Bioresources and Bioprocessing.

[b1160] Zhao Y.D., Shi C.C., Su Q.X., Pei D., Huang X.Y. (2025). Research progress on extraction and isolation, biosynthesis, biological activities of astragalosides and its application prospects. Chinese Herbal Medicines.

[b1165] Zhao D.D., Feng X., Zhu X.N., Wu T., Zhang X.L., Bi C.H. (2017). CRISPR/Cas9-assisted gRNA-free one-step genome editing with no sequence limitations and improved targeting efficiency. Scientific Reports.

[b1170] Zhao D.D., Zhu X.N., Zhou H., Sun N.X., Wang T., Bi C.H. (2021). CRISPR-based metabolic pathway engineering. Metabolic Engineering.

[b1175] Zhao Y., Coelho C., Hughes A.L., Lazar-Stefanita L., Yang S., Brooks A.N. (2023). Debugging and consolidating multiple synthetic chromosomes reveals combinatorial genetic interactions. Cell.

[b1180] Zhong Z.X., Zhou S., Liang Y.J., Wei Y.Y., Li Y., Long T.F. (2023). Natural flavonoids disrupt bacterial iron homeostasis to potentiate colistin efficacy. Science Advances.

[b1185] Zhou C., Chen T.J., Gu A.D., Hu Z.F., Li Y., Gong T. (2023). Combining protein and metabolic engineering to achieve green biosynthesis of 12*β*-*O*-Glc-PPD in *Saccharomyces cerevisiae*. Green Chemistry.

[b1195] Zhou S.H., Hao T.T., Zhou J.W. (2020). Fermentation and metabolic pathway optimization to *de novo* synthesize (2*S*)-naringenin in *Escherichia coli*. Journal of Microbiology and Biotechnology.

[b1200] Zhou T.A., Park Y.K., Fu J., Hapeta P., Klemm C., Ledesma-Amaro R. (2025). Metabolic engineering of *Yarrowia lipolytica* for the production and secretion of the saffron ingredient crocetin. Biotechnology for Biofuels and Bioproducts.

[b1205] Zhou X.J., Zhang X.X., Wang D., Luo R.S., Qin Z., Lin F.Z. (2024). Efficient biosynthesis of salidroside via artificial *in vivo* enhanced UDP-glucose system using cheap sucrose as substrate. ACS Omega.

[b1210] Zhu H.J., Kerčmar P., Wu F.R., Rajendran C., Sun L.L., Wang M.T. (2015). Using strictosidine synthase to prepare novel alkaloids. Current Medicinal Chemistry.

[b1215] Zhu J.W., Zhang K., He Y.Z., Zhang Q., Ran Y.P., Tan Z.G. (2024). Metabolic engineering of *Saccharomyces cerevisiae* for chelerythrine biosynthesis. Microbial Cell Factories.

[b1220] Zhuang W.B., Li Y.H., Shu X.C., Pu Y.T., Wang X.J., Wang T. (2023). The classification, molecular structure and biological biosynthesis of flavonoids, and their roles in biotic and abiotic stresses. Molecules.

[b1225] Zou Y.S., Li C.Y., Zhang R.H., Jiang T., Liu N., Wang J. (2021). Exploring the tunability and dynamic properties of MarR-PmarO sensor system in *Escherichia coli*. ACS Synthetic Biology.

[b1230] Zwenger S., Basu C. (2008). Plant terpenoids: Applications and future potentials. Biotechnology and Molecular Biology Reviews.

